# A Multi-Parameter Perturbation Solution for Functionally Graded Piezoelectric Cantilever Beams under Combined Loads

**DOI:** 10.3390/ma11071222

**Published:** 2018-07-16

**Authors:** Yongsheng Lian, Xiaoting He, Sijie Shi, Xue Li, Zhixin Yang, Junyi Sun

**Affiliations:** 1 School of Civil Engineering, Chongqing University, Chongqing 400045, China; lianyongsheng@cqu.edu.cn (Y.L.); shisj@yahoo.com (S.S.); lixuecqu@126.com (X.L.); yangzhixin123@126.com (Z.Y.); sunjunyi@cqu.edu.cn (J.S.); 2 Key Laboratory of New Technology for Construction of Cities in Mountain Area (Chongqing University), Ministry of Education, Chongqing 400045, China

**Keywords:** functionally graded piezoelectric materials, cantilever beams, multi-parameter perturbation method, piezoelectric coefficients

## Abstract

In this study, we use a multi-parameter perturbation method to solve the problem of a functionally graded piezoelectric cantilever beam under combined loads, in which three piezoelectric coefficients are selected as the perturbation parameters. First, we derive the two basic equations concerning the Airy stress function and electric potential function. By expanding the unknown Airy stress function and electric potential function with respect to three perturbation parameters, the two basic equations were decoupled, thus obtaining the corresponding multi-parameter perturbation solution under boundary conditions. From the solution obtained, we can see clearly how the piezoelectric effects influence the behavior of the functionally graded piezoelectric cantilever beam. Based on a numerical example, the variations of the elastic stresses and displacements as well as the electric displacements of the cantilever beam under different gradient exponents were shown. The results indicate that if the pure functionally graded cantilever beam without a piezoelectric effect is regarded as an unperturbed system, the functionally graded piezoelectric cantilever beam can be looked upon as a perturbed system, thus opening the possibilities for perturbation solving. Besides, the proposed multi-parameter perturbation method provides a new idea for solving similar nonlinear differential equations.

## 1. Introduction

Functionally graded piezoelectric materials (FGPMs) have been increasingly used in piezoelectric sensors and actuators [1,2]. The FGPMs inherit the advantages of functionally graded materials (FGMs) and piezoelectric materials. The FGMs consist of two or more materials in which the composition of the materials varies continuously in certain directions, and there is no obvious interface in FGMs [3]. Therefore, the stress concentration problem caused by the bonding of the two materials can be avoided by using FGMs. The advantage of piezoelectric materials is their good conversion ability between mechanical energy and electric energy. Piezoelectricity is very suitable for physical sensors and biosensors construction [4] and there are many valuable applications in engineering fields (for example, structural health monitoring [5]). Piezoelectric materials characterization is a challenging problem involving physical concepts, electrical and mechanical measurements, and numerical optimization techniques [6,7]. Thus, the analysis of piezoelectric materials and structures becomes more and more important. However, the difficulties in studying FGMs and piezoelectric materials are also inherited by FGPMs, and the nonlinear differential governing equations of the FGPM structures are usually difficult to be analytically solved.

Over the past few decades, researchers have devoted a lot of effort to the problems of FGMs and FGPMs and have harvested some fruits. Eshraghi et al. [8]. studied the bending and free vibrations of FGM annular and circular micro-plates under thermal loading. Kim and Reddy [9] derived the equations of motion for FGM plates with surface-mounted piezoelectric layers by using Hamilton’s principle, in which the gradient elasticity was accounted for through the modified couple stress model and linear piezoelectricity. Kahya and Turan [10] presented a finite element model for free vibration and buckling analyses of FGM sandwich beams on the basis of first-order shear deformation theory. By using Hamilton’s variational principle and the classical plate theory, Arshid and Khorshidvand [11] studied the free vibration analysis of saturated porous FGM circular plates integrated by piezoelectric actuator patches via a differential quadrature method. On the basis of classical plate theory, Żur presented the analysis and numerical results for the free axisymmetric and non-axisymmetric vibrations of FGM circular plates elastically supported on a concentric ring [12] and annular plates elastically supported on the ring support [13] via quasi-Green’s function method. Zhu et al. [14,15] originally introduced the concept of FGMs into piezoelectric materials, and successfully manufactured FGPM actuators. Shi et al. presented the solution of FGPM cantilever beams subjected to different loadings [16], and investigated the electrostatic behavior of piezoelectric cantilevers with a nonlinear piezoelectric parameter [17]. Huang et al. proposed a piezoelasticity solution for FGPM cantilever beams under different loading conditions [18] and a unified solution for an anisotropic FGPM cantilever beam subject to sinusoidal transverse loads [19]. Zhong and Yu obtained a solution for FGPM cantilever beams under different loadings by assuming that the mechanical and electrical properties of the material have the same variations along the thickness direction [20], and proposed a general solution for FGPM cantilever beams with arbitrary graded material properties along the beam thickness direction by expressing the Airy stress function and the electric potential function in finite power series [21]. Yang and Xiang [22] and Komeili et al. [23] investigated the static bending FGPM beams under combined thermo-electro-mechanical loads. Based on the modified strain gradient theory and Timoshenko beam theory, Li et al. [24] developed a size-dependent FGPM beam model by using variational formulation, and solved the static bending and free vibration problems of a simply supported FGPM beam. Lin and Muliana [25] studied the nonlinear electro-mechanical responses of FGPM beams undergoing small deformation gradients. Pandey and Parashar [26] investigated the static bending of the FGPM beam under electromechanical loading, in which the effective material properties of the FGPM beam are graded according to sigmoid law distribution. Duc et al. [27] investigated the nonlinear dynamic response and vibration of an eccentrically stiffened FGPM plate subjected to mechanical and electrical loads in a thermal environment. Su et al. [28] dealt with the electro-mechanical vibration characteristics of FGPM rectangular plates with different boundary conditions based on first-order shear deformation theory. More recently, He et al. [29] presented an electroelastic solution for FGPM beams with different moduli in tension and compression. Given that there are many relative works in this field, we do not review them in detail.

From the above studies, we may see that in the analysis of FGPM beams, the number of basic equations used for the solution of the problem is so large that it is difficult to solve them analytically; at least the process is relatively complex. In addition, the basic equations are generally presented in the form of a high-order partial differential equation, which further aggravates the complexity of the solution. For this purpose, we need to seek an effective mathematical method for similar boundary value problems. 

The parametric perturbation method (PPM) proposed by Poincaré [30] is one of the standard analytical methods used for the solution of nonlinear problems in applied mechanics and physics. Many studies have indicated that this method is a general analytical method for obtaining approximate solutions of nonlinear differential equations in initial or boundary value problems. In PPM, the solution of the nonlinear differential equation is constructed by developing an asymptotic series with respect to a certain parameter. The so-called perturbation is generated in the neighborhood of the solution of the unperturbed equation, so that the known properties of the unperturbed linear system can be used to obtain the solution of the perturbed system. More recently, this basic idea of perturbation was demonstrated again by Lian et al. [31], in which the Hencky membrane problem without a small-rotation-angle assumption was solved by perturbation to the corresponding classical small-rotation-angle problem. Originally, there was only a single perturbation parameter in the PPM, which was called the single-parameter perturbation method (S-PPM), and many classical works were based on the PPM. Later, as the method was continuously studied, scholars began to discover if multiple parameters are introduced, the perturbation solution characterized by these parameters may well describe the separate influence of each parameter on the nonlinearity of the problem. The earlier work can be seen from Nowinski and Ismail [32], in which a multi-parameter perturbation method (M-PPM) was proposed to solve the deformation problem of a cylindrical orthotropic circular plate. The pioneer work in nonlinear beam problems was done by Chien [33], in which a biparametric perturbation method (B-PPM) was initially applied to solve the classical Euler-Bernoulli equation of beams with a height difference between the two ends from a practical engineering problem. Later, He et al. successfully used the so-called B-PPM to solve large deflection beam problems which Chien dealt with [34] and large deflection circular plate problems with a bimodular effect [35]. However, the application of the real M-PPM which contains three or more perturbation parameters has not been found yet.

In this study, we extended the traditional S-PPM and B-PPM to M-PPM which contains three perturbation parameters and solved the governing equations of the FGPM cantilever beam under combined loads. The piezoelectric coefficients are selected as perturbation parameters. Thus, from the point of view of the perturbation idea, if the pure FGM cantilever beam is regarded as an unperturbed system, the FGPM cantilever beam can be looked upon as a perturbed system. In the next section, the mechanical model of a FGPM cantilever beam under the combined action of a uniformly distributed load, concentrated force, and bending moment is presented. In Section 3, the perturbation solution of the FGPM cantilever beam is obtained. In Section 4, based on a numerical example, the variations of the elastic stresses and displacements, as well as the electric displacements, are shown and some important issues are discussed. Section 5 is the concluding remarks.

## 2. Mechanical Model and Basic Equations

**Figure 1:**
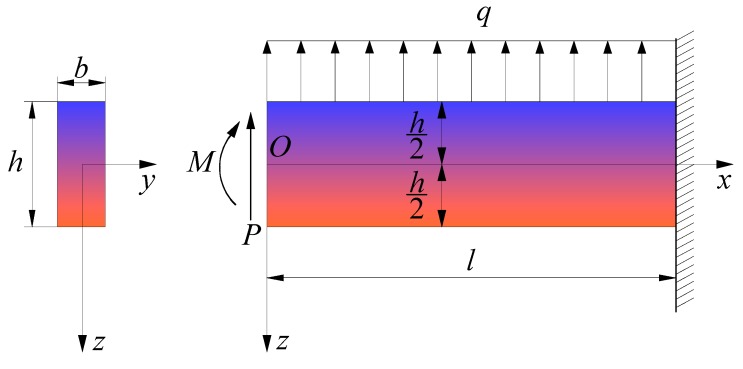
Scheme of a functionally graded piezoelectric cantilever beam.

In this study, the mechanical model of the FGPM cantilever beam is established by using two-dimensional elastic beam theory and neglecting shear deformation, since what we consider here is a relatively shallow beam. Generally speaking, the mechanical and electrical parameters of FGPMs change along one direction only. In this study, we assume that the mechanical and electrical parameters of the FGPM cantilever beam vary along the thickness of the FGPM cantilever beam. As shown in Figure 1, an FGPM cantilever beam is fixed at its right end and subjected to uniformly distributed loads *q* on its upper surface, a concentrated force *P*, and a bending moment *M* at its left end, in which *l*, *b*, and *h* (*h << l*) denote the length, width, and height of the beam, respectively. A rectangular coordinate system is introduced with the upper and lower surfaces of the beam lying in z=−h/2 and z=h/2. The mechanical and electrical parameters of the FGPM cantilever beam vary along the *z* coordinate, such that

(1) $$s_{ij}=s_{ij}^{0}e^{\alpha z/h},d_{ij}=d_{ij}^{0}e^{\alpha z/h},\lambda_{ij}=\lambda_{ij}^{0}e^{\alpha z/h},$$

where *α* is a gradient exponent; $s_{ij}^{}$, $d_{ij}^{}$, and $\lambda_{ij}^{}$ are the elastic coefficient, piezoelectric coefficient, and dielectric coefficient, respectively; and $s_{ij}^{0}$, $d_{ij}^{0}$, and $\lambda_{ij}^{0}$ are values of the corresponding material parameters at z=0, respectively.

By neglecting body forces and free charges, the mechanical equation of equilibrium and the electrical equation of equilibrium are

(2) $$\left\{ \begin{array}{l} \frac{\partial \sigma_{x}}{\partial x}+\frac{\partial \tau_{zx}}{\partial z}=0\\ \frac{\partial \tau_{zx}}{\partial x}+\frac{\partial \sigma_{z}}{\partial z}=0 \end{array}\right.$$

and

(3) $$\frac{\partial D_{x}}{\partial x}+\frac{\partial D_{z}}{\partial z}=0,$$

where $\sigma_{x}$, $\sigma_{z}$, and $\tau_{zx}$ are the stress components; and $D_{x}$ and $D_{z}$ are the electric displacement components. The constitutive equations of the materials are

(4) $$\left\{ \begin{array}{l} \varepsilon_{x}=s_{11}\sigma_{x}+s_{13}\sigma_{z}+d_{31}E_{z}\\ \varepsilon_{z}=s_{13}\sigma_{x}+s_{33}\sigma_{z}+d_{33}E_{z}\\ \gamma_{zx}=s_{44}\tau_{zx}+d_{15}E_{x}\\ D_{x}=d_{15}\tau_{zx}+\lambda_{11}E_{x}\\ D_{z}=d_{31}\sigma_{x}+d_{33}\sigma_{z}+\lambda_{33}E_{z} \end{array}\right.,$$

where $\varepsilon_{x}$, $\varepsilon_{z}$, and $\gamma_{zx}$ are the strain components; and $E_{x}$ and $E_{z}$ are the electric field components. The geometric equations give

(5) $$\varepsilon_{x}=\frac{\partial u}{\partial x},\varepsilon_{z}=\frac{\partial w}{\partial z},\gamma_{zx}=\frac{\partial u}{\partial z}+\frac{\partial w}{\partial x},$$

where *u* and *w* are the displacement components. The strain compatibility equation is

(6) $$\frac{\partial^{2}\varepsilon_{x}}{\partial z^{2}}+\frac{\partial^{2}\varepsilon_{z}}{\partial x^{2}}-\frac{\partial^{2}\gamma_{zx}}{\partial z\partial x}=0.$$

The relationships between the electric field components and the electric potential are

(7) $$E_{x}=-\frac{\partial \Phi }{\partial x},\;E_{z}=-\frac{\partial \Phi }{\partial z},$$

where Φ is the electric potential function. By introducing Airy stress function *U*(*x*, *z*), we may express the stress components as

(8) $$\sigma_{x}=\frac{\partial^{2}U}{\partial z^{2}},\;\sigma_{z}=\frac{\partial^{2}U}{\partial x^{2}},\;\tau_{zx}=-\frac{\partial^{2}U}{\partial z\partial x}.$$

Substituting Equations (4), (7), and (8) into Equations (3) and (6), the governing equations for the Airy stress function *U*(*x*, *z*) and the electric potential function Φ(*x*, *z*) are

(9) $$\frac{\partial }{\partial z}(d_{31}\frac{\partial^{2}U}{\partial z^{2}})+\frac{\partial }{\partial z}(d_{33}\frac{\partial^{2}U}{\partial x^{2}})-d_{15}\frac{\partial^{3}U}{\partial x^{2}\partial z}=\frac{\partial }{\partial z}(\lambda_{33}\frac{\partial \Phi }{\partial z})+\lambda_{11}\frac{\partial^{2}\Phi }{\partial x^{2}}$$

and

(10) $$\begin{array}{l} \frac{\partial^{2}}{\partial z^{2}}(s_{11}\frac{\partial^{2}U}{\partial z^{2}}+s_{13}\frac{\partial^{2}U}{\partial x^{2}})+\frac{\partial }{\partial z}(s_{44}\frac{\partial^{3}U}{\partial x^{2}\partial z})+s_{13}\frac{\partial^{4}U}{\partial x^{2}\partial z^{2}}+s_{33}\frac{\partial^{4}U}{\partial x^{4}}\\ =\frac{\partial^{2}}{\partial z^{2}}(d_{31}\frac{\partial \Phi }{\partial z})+d_{33}\frac{\partial^{3}\Phi }{\partial x^{2}\partial z}-\frac{\partial }{\partial z}(d_{15}\frac{\partial^{2}\Phi }{\partial x^{2}}) \end{array},$$

where $d_{31}^{0}$, $d_{33}^{0}$, and $d_{15}^{0}$ in the piezoelectric coefficients $d_{31}^{}$, $d_{33}^{}$, and $d_{15}^{}$ may be selected as the perturbation parameters. When $d_{31}^{0}=d_{33}^{0}=d_{15}^{0}=0$, Equation (10) may be regressed into the governing equation of the pure functionally graded cantilever beam (Equation (11) in [36]), i.e.,

(11) $$\frac{\partial^{2}}{\partial z^{2}}(s_{11}\frac{\partial^{2}U}{\partial z^{2}}+s_{13}\frac{\partial^{2}U}{\partial x^{2}})+\frac{\partial }{\partial z}(s_{44}\frac{\partial^{3}U}{\partial x^{2}\partial z})+s_{13}\frac{\partial^{4}U}{\partial x^{2}\partial z^{2}}+s_{33}\frac{\partial^{4}U}{\partial x^{4}}=0.$$

The mechanical and electrical boundary conditions are given as follows:

(12) $$\int_{-h/2}^{h/2}\tau_{zx}dz=\frac{P}{b},\;\int_{-h/2}^{h/2}\sigma_{x}dz=0\;\mathrm{and}\;\int_{-h/2}^{h/2}z\sigma_{x}dz=\frac{M}{b},\;\mathrm{at}\;x=0,$$

(13) $$\left\{ \begin{array}{l} \sigma_{z}=\tau_{zx}=0,\;\mathrm{at}\;z=h/2\\ \sigma_{z}=q,\;\tau_{zx}=0,\;\mathrm{at}\;z=-h/2 \end{array}\right.,$$

(14) $$\left\{ \begin{array}{l} \int_{-h/2}^{h/2}D_{x}dz=0,\;\mathrm{at}\;x=0\;\mathrm{and}\;x=l\\ D_{z}=0,\;\mathrm{at}\;z=h/2\;\mathrm{and}\;z=-h/2 \end{array}\right.$$

and

(15) $$u=w=\frac{\partial w}{\partial x}=0,\;\mathrm{at}\;z=0\;\mathrm{and}\;x=l$$

## 3. Perturbation Solution

Substituting Equation (1) into Equations (9) and (10), we have

(16) $$\left\{ \begin{array}{l} d_{31}^{0}\frac{\alpha }{h}\frac{\partial^{2}U}{\partial z^{2}}+d_{33}^{0}\frac{\alpha }{h}\frac{\partial^{2}U}{\partial x^{2}}+d_{31}^{0}\frac{\partial^{3}U}{\partial z^{3}}+(d_{33}^{0}-d_{15}^{0})\frac{\partial^{3}U}{\partial x^{2}\partial z}=\lambda_{33}^{0}\frac{\alpha }{h}\frac{\partial \Phi }{\partial z}+\lambda_{33}^{0}\frac{\partial^{2}\Phi }{\partial z^{2}}+\lambda_{11}^{0}\frac{\partial^{2}\Phi }{\partial x^{2}}\\ s_{11}^{0}\frac{\alpha^{2}}{h^{2}}\frac{\partial^{2}U}{\partial z^{2}}+s_{13}^{0}\frac{\alpha^{2}}{h^{2}}\frac{\partial^{2}U}{\partial x^{2}}+2s_{11}^{0}\frac{\alpha }{h}\frac{\partial^{3}U}{\partial z^{3}}+(2s_{13}^{0}+s_{44}^{0})\frac{\alpha }{h}\frac{\partial^{3}U}{\partial x^{2}\partial z}+s_{11}^{0}\frac{\partial^{4}U}{\partial z^{4}}+s_{33}^{0}\frac{\partial^{4}U}{\partial x^{4}}\\ +(s_{44}^{0}+2s_{13}^{0})\frac{\partial^{4}U}{\partial x^{2}\partial z^{2}}=d_{31}^{0}\frac{\alpha^{2}}{h^{2}}\frac{\partial \Phi }{\partial z}+2d_{31}^{0}\frac{\alpha }{h}\frac{\partial^{2}\Phi }{\partial z^{2}}+d_{31}^{0}\frac{\partial^{3}\Phi }{\partial z^{3}}+(d_{33}^{0}-d_{15}^{0})\frac{\partial^{3}\Phi }{\partial x^{2}\partial z}-d_{15}^{0}\frac{\alpha }{h}\frac{\partial^{2}\Phi }{\partial x^{2}} \end{array}.\right.$$

From the piezoelectric parameters of the five kinds of piezoelectric materials listed by Ruan et al. [37], it can be seen that the piezoelectric coefficients are usually very small. So, they can be selected as perturbation parameters to meet the requirement of convergence in perturbation expansions. Thus, from the point of view of the perturbation idea, if the pure FGM cantilever beam is regarded as an unperturbed system, the FGPM cantilever beam can be looked upon as a perturbed system. By selecting $d_{31}^{0}$, $d_{33}^{0}$, and $d_{15}^{0}$ as perturbation parameters, we may expand Φ and *U* with respect to $d_{31}^{0}$, $d_{33}^{0}$, and $d_{15}^{0}$, as follows:

(17) $$\begin{array}{l} \Phi =\Phi_{0}^{0}+\Phi_{1}^{I}d_{31}^{0}+\Phi_{2}^{I}d_{33}^{0}+\Phi_{3}^{I}d_{15}^{0}+\Phi_{1}^{\mathrm{II}}{\left(d_{31}^{0}\right)}^{2}+\Phi_{2}^{\mathrm{II}}{\left(d_{33}^{0}\right)}^{2}\\ \;+\Phi_{3}^{\mathrm{II}}{\left(d_{15}^{0}\right)}^{2}+\Phi_{4}^{\mathrm{II}}d_{31}^{0}d_{33}^{0}+\Phi_{5}^{\mathrm{II}}d_{31}^{0}d_{15}^{0}+\Phi_{6}^{\mathrm{II}}d_{33}^{0}d_{15}^{0} \end{array}$$

and

(18) $$\begin{array}{l} U=U_{0}^{0}+U_{1}^{I}d_{31}^{0}+U_{2}^{I}d_{33}^{0}+U_{3}^{I}d_{15}^{0}+U_{1}^{\mathrm{II}}{\left(d_{31}^{0}\right)}^{2}+U_{2}^{\mathrm{II}}{\left(d_{33}^{0}\right)}^{2}\\ \;+U_{3}^{\mathrm{II}}{\left(d_{15}^{0}\right)}^{2}+U_{4}^{\mathrm{II}}d_{31}^{0}d_{33}^{0}+U_{5}^{\mathrm{II}}d_{31}^{0}d_{15}^{0}+U_{6}^{\mathrm{II}}d_{33}^{0}d_{15}^{0} \end{array},$$

where $\Phi_{0}^{0}$ and $U_{0}^{0}$, $\Phi_{i}^{I}$ and $U_{i}^{I}$ (i=1,2,3), and $\Phi_{i}^{\mathrm{II}}$ and $U_{i}^{\mathrm{II}}$(i=1,2,3,...,6) are unknown functions of *x* and *z*.

Substituting Equations (17) and (18) into Equation (16), as well as into the boundary conditions, Equations (12)–(14), we may obtain a series of decomposed differential equations and the corresponding boundary conditions by comparing the coefficients of the same power of $d_{31}^{0}$, $d_{33}^{0}$, and $d_{15}^{0}$.

1. By comparing the coefficients of ${\left(d_{31}^{0}\right)}^{0}$, ${\left(d_{33}^{0}\right)}^{0}$, and ${\left(d_{15}^{0}\right)}^{0}$ in Equation (16), we may obtain the differential equations for $\Phi_{0}^{0}$ and $U_{0}^{0}$,

(19) $$\left\{ \begin{array}{l} \lambda_{33}^{0}\frac{\alpha }{h}\frac{\partial \Phi_{0}^{0}}{\partial z}+\lambda_{33}^{0}\frac{\partial^{2}\Phi_{0}^{0}}{\partial z^{2}}+\lambda_{11}^{0}\frac{\partial^{2}\Phi_{0}^{0}}{\partial x^{2}}=0\\ s_{11}^{0}\frac{\alpha^{2}}{h^{2}}\frac{\partial^{2}U_{0}^{0}}{\partial z^{2}}+s_{13}^{0}\frac{\alpha^{2}}{h^{2}}\frac{\partial^{2}U_{0}^{0}}{\partial x^{2}}+2s_{11}^{0}\frac{\alpha }{h}\frac{\partial^{3}U_{0}^{0}}{\partial z^{3}}+(2s_{13}^{0}+s_{44}^{0})\frac{\alpha }{h}\frac{\partial^{3}U_{0}^{0}}{\partial x^{2}\partial z}\\ +s_{11}^{0}\frac{\partial^{4}U_{0}^{0}}{\partial z^{4}}+s_{33}^{0}\frac{\partial^{4}U_{0}^{0}}{\partial x^{4}}+(s_{44}^{0}+2s_{13}^{0})\frac{\partial^{4}U_{0}^{0}}{\partial x^{2}\partial z^{2}}=0 \end{array}\right.,$$

which may be solved under the boundary conditions

(20) $$\int_{-h/2}^{h/2}(-\frac{\partial^{2}U_{0}^{0}}{\partial z\partial x})dz=\frac{P}{b},\int_{-h/2}^{h/2}\frac{\partial^{2}U_{0}^{0}}{\partial z^{2}}dz=0\;\mathrm{and}\;\int_{-h/2}^{h/2}z\frac{\partial^{2}U_{0}^{0}}{\partial z^{2}}dz=\frac{M}{b},\;\mathrm{at}\;x=0$$

(21) $$\left\{ \begin{array}{l} \frac{\partial^{2}U_{0}^{0}}{\partial x^{2}}=-\frac{\partial^{2}U_{0}^{0}}{\partial z\partial x}=0,\;\mathrm{at}\;z=h/2\\ \frac{\partial^{2}U_{0}^{0}}{\partial x^{2}}=q,\;-\frac{\partial^{2}U_{0}^{0}}{\partial z\partial x}=0,\;\mathrm{at}\;z=-h/2 \end{array}\right.$$

and

(22) $$\left\{ \begin{array}{l} \int_{-h/2}^{h/2}(-d_{15}\frac{\partial^{2}U_{0}^{0}}{\partial z\partial x}-\lambda_{11}\frac{\partial \Phi_{0}^{0}}{\partial x})dz=0,\;\mathrm{at}\;x=0\;\mathrm{and}\;x=l\\ d_{31}\frac{\partial^{2}U_{0}^{0}}{\partial z^{2}}+d_{33}\frac{\partial^{2}U_{0}^{0}}{\partial x^{2}}-\lambda_{33}\frac{\partial \Phi_{0}^{0}}{\partial z}=0,\;\mathrm{at}\;z=h/2\;\mathrm{and}\;z=-h/2 \end{array}\right..$$

Suppose

(23) $$\left\{ \begin{array}{l} \Phi_{0}^{0}=x^{2}g_{1}^{0}(z)+xg_{2}^{0}(z)+g_{3}^{0}(z)\\ U_{0}^{0}=\frac{x^{2}}{2}f_{1}^{0}(z)+xf_{2}^{0}(z)+f_{3}^{0}(z) \end{array}\right.,$$

where $g_{i}^{0}(z)$ and $f_{i}^{0}(z)$ (i=1,2,3) are unknown functions of *z*. After Substituting Equation (23) into Equation (19), it is found that

(24) $$\left\{ \begin{array}{l} g_{1}^{0}(z)=B_{1}^{0}+B_{2}^{0}e^{-\frac{\alpha }{h}z}\\ g_{2}^{0}(z)=B_{3}^{0}+B_{4}^{0}e^{-\frac{\alpha }{h}z}\\ g_{3}^{0}(z)=B_{5}^{0}+B_{6}^{0}e^{-\frac{\alpha }{h}z}-2\frac{h}{\alpha }\frac{\lambda_{11}^{0}}{\lambda_{33}^{0}}B_{1}^{0}z+2\frac{h}{\alpha }\frac{\lambda_{11}^{0}}{\lambda_{33}^{0}}B_{2}^{0}ze^{-\frac{\alpha }{h}z} \end{array}\right.$$

and

(25) $$\left\{ \begin{array}{l} f_{1}^{0}(z)=C_{1}^{0}+C_{2}^{0}z+(C_{3}^{0}+C_{4}^{0}z)e^{-\frac{\alpha }{h}z}\\ f_{2}^{0}(z)=C_{5}^{0}+C_{6}^{0}z+(C_{7}^{0}+C_{8}^{0}z)e^{-\frac{\alpha }{h}z}\\ f_{3}^{0}(z)=C_{9}^{0}+C_{10}^{0}z+(C_{11}^{0}+C_{12}^{0}z)e^{-\frac{\alpha }{h}z}-\frac{z^{2}}{6s_{11}^{0}}(3s_{13}^{0}C_{1}^{0}+3s_{44}^{0}\frac{h}{\alpha }C_{2}^{0}+s_{13}^{0}C_{2}^{0}z)\\ \;-\frac{z^{2}}{6s_{11}^{0}}(3s_{13}^{0}C_{3}^{0}-3s_{44}^{0}\frac{h}{\alpha }C_{4}^{0}+s_{13}^{0}C_{4}^{0}z)e^{-\frac{\alpha }{h}z} \end{array}\right.,$$

where $B_{i}^{0}$ (i=1,2,3,...,6) and $C_{i}^{0}$ (i=1,2,3,...,12) are undetermined constants which can be determined by Equations (20)–(22), please see Appendix A.

2. Similarly, by comparing the coefficients of ${\left(d_{31}^{0}\right)}_{}^{1}$, ${\left(d_{33}^{0}\right)}_{}^{1}$, and ${\left(d_{15}^{0}\right)}_{}^{1}$ in Equation (16), we may obtain the differential equations for $\Phi_{i}^{I}$ and $U_{i}^{I}$ (i=1,2,3), respectively, for term ${\left(d_{31}^{0}\right)}_{}^{1}$:

(26) $$\left\{ \begin{array}{l} \lambda_{33}^{0}\frac{\alpha }{h}\frac{\partial \Phi_{1}^{I}}{\partial z}+\lambda_{33}^{0}\frac{\partial^{2}\Phi_{1}^{I}}{\partial z^{2}}+\lambda_{11}^{0}\frac{\partial^{2}\Phi_{1}^{I}}{\partial x^{2}}=\frac{\alpha }{h}\frac{\partial^{2}U_{0}^{0}}{\partial z^{2}}+\frac{\partial^{3}U_{0}^{0}}{\partial z^{3}}\\ s_{11}^{0}\frac{\alpha^{2}}{h^{2}}\frac{\partial^{2}U_{1}^{I}}{\partial z^{2}}+s_{13}^{0}\frac{\alpha^{2}}{h^{2}}\frac{\partial^{2}U_{1}^{I}}{\partial x^{2}}+2s_{11}^{0}\frac{\alpha }{h}\frac{\partial^{3}U_{1}^{I}}{\partial z^{3}}+(2s_{13}^{0}+s_{44}^{0})\frac{\alpha }{h}\frac{\partial^{3}U_{1}^{I}}{\partial x^{2}\partial z}+s_{11}^{0}\frac{\partial^{4}U_{1}^{I}}{\partial z^{4}}\\ +s_{33}^{0}\frac{\partial^{4}U_{1}^{I}}{\partial x^{4}}+(s_{44}^{0}+2s_{13}^{0})\frac{\partial^{4}U_{1}^{I}}{\partial x^{2}\partial z^{2}}=\frac{\alpha^{2}}{h^{2}}\frac{\partial \Phi_{0}^{0}}{\partial z}+2\frac{\alpha }{h}\frac{\partial^{2}\Phi_{0}^{0}}{\partial z^{2}}+\frac{\partial^{3}\Phi_{0}^{0}}{\partial z^{3}} \end{array},\right.$$

for term ${\left(d_{33}^{0}\right)}_{}^{1}$:

(27) $$\left\{ \begin{array}{l} \lambda_{33}^{0}\frac{\alpha }{h}\frac{\partial \Phi_{2}^{I}}{\partial z}+\lambda_{33}^{0}\frac{\partial^{2}\Phi_{2}^{I}}{\partial z^{2}}+\lambda_{11}^{0}\frac{\partial^{2}\Phi_{2}^{I}}{\partial x^{2}}=\frac{\alpha }{h}\frac{\partial^{2}U_{0}^{0}}{\partial x^{2}}+\frac{\partial^{3}U_{0}^{0}}{\partial x^{2}\partial z}\\ s_{11}^{0}\frac{\alpha^{2}}{h^{2}}\frac{\partial^{2}U_{2}^{I}}{\partial z^{2}}+s_{13}^{0}\frac{\alpha^{2}}{h^{2}}\frac{\partial^{2}U_{2}^{I}}{\partial x^{2}}+2s_{11}^{0}\frac{\alpha }{h}\frac{\partial^{3}U_{2}^{I}}{\partial z^{3}}+(2s_{13}^{0}+s_{44}^{0})\frac{\alpha }{h}\frac{\partial^{3}U_{2}^{I}}{\partial x^{2}\partial z}\\ +s_{11}^{0}\frac{\partial^{4}U_{2}^{I}}{\partial z^{4}}+s_{33}^{0}\frac{\partial^{4}U_{2}^{I}}{\partial x^{4}}+(s_{44}^{0}+2s_{13}^{0})\frac{\partial^{4}U_{2}^{I}}{\partial x^{2}\partial z^{2}}=\frac{\partial^{3}\Phi_{0}^{0}}{\partial x^{2}\partial z} \end{array}\right.,$$

and for term ${\left(d_{15}^{0}\right)}_{}^{1}$:

(28) $$\left\{ \begin{array}{l} \lambda_{33}^{0}\frac{\alpha }{h}\frac{\partial \Phi_{3}^{I}}{\partial z}+\lambda_{33}^{0}\frac{\partial^{2}\Phi_{3}^{I}}{\partial z^{2}}+\lambda_{11}^{0}\frac{\partial^{2}\Phi_{3}^{I}}{\partial x^{2}}=-\frac{\partial^{3}U_{0}^{0}}{\partial x^{2}\partial z}\\ s_{11}^{0}\frac{\alpha^{2}}{h^{2}}\frac{\partial^{2}U_{3}^{I}}{\partial z^{2}}+s_{13}^{0}\frac{\alpha^{2}}{h^{2}}\frac{\partial^{2}U_{3}^{I}}{\partial x^{2}}+2s_{11}^{0}\frac{\alpha }{h}\frac{\partial^{3}U_{3}^{I}}{\partial z^{3}}+(2s_{13}^{0}+s_{44}^{0})\frac{\alpha }{h}\frac{\partial^{3}U_{3}^{I}}{\partial x^{2}\partial z}\\ +s_{11}^{0}\frac{\partial^{4}U_{3}^{I}}{\partial z^{4}}+s_{33}^{0}\frac{\partial^{4}U_{3}^{I}}{\partial x^{4}}+(s_{44}^{0}+2s_{13}^{0})\frac{\partial^{4}U_{3}^{I}}{\partial x^{2}\partial z^{2}}=-\frac{\partial^{3}\Phi_{0}^{0}}{\partial x^{2}\partial z}-\frac{\alpha }{h}\frac{\partial^{2}\Phi_{0}^{0}}{\partial x^{2}} \end{array},\right.$$

which may be solved under the boundary conditions

(29) $$\int_{-h/2}^{h/2}(-\frac{\partial^{2}U_{i}^{I}}{\partial z\partial x})dz=0,\;\int_{-h/2}^{h/2}\frac{\partial^{2}U_{i}^{I}}{\partial z^{2}}dz=0\;\mathrm{and}\;\int_{-h/2}^{h/2}z\frac{\partial^{2}U_{i}^{I}}{\partial z^{2}}dz=0\;\left(i=1,2,3\right),\;\mathrm{at}\;x=0,$$

(30) $$\left\{ \begin{array}{l} \frac{\partial^{2}U_{i}^{I}}{\partial x^{2}}=-\frac{\partial^{2}U_{i}^{I}}{\partial z\partial x}=0,\;\mathrm{at}\;z=h/2\\ \frac{\partial^{2}U_{i}^{I}}{\partial x^{2}}=-\frac{\partial^{2}U_{i}^{I}}{\partial z\partial x}=0,\;\mathrm{at}\;z=-h/2 \end{array}\right.\left(i=1,2,3\right)$$

and

(31) $$\left\{ \begin{array}{l} \int_{-h/2}^{h/2}(-d_{15}\frac{\partial^{2}U_{i}^{I}}{\partial z\partial x}-\lambda_{11}\frac{\partial \Phi_{i}^{I}}{\partial x})dz=0,\;\mathrm{at}\;x=0\;\mathrm{and}\;x=l\\ d_{31}\frac{\partial^{2}U_{i}^{I}}{\partial z^{2}}+d_{33}\frac{\partial^{2}U_{i}^{I}}{\partial x^{2}}-\lambda_{33}\frac{\partial \Phi_{i}^{I}}{\partial z}=0,\;\mathrm{at}\;z=h/2\;\mathrm{and}\;z=-h/2 \end{array}\right.\left(i=1,2,3\right).$$

Suppose

(32) $$\left\{ \begin{array}{l} \Phi_{i}^{I}=x^{2}g_{3i-2}^{I}(z)+xg_{3i-1}^{I}(z)+g_{3i}^{I}(z)\\ U_{i}^{I}=\frac{x^{2}}{2}f_{3i-2}^{I}(z)+xf_{3i-1}^{I}(z)+f_{3i}^{I}(z) \end{array}\right.\;\left(i=1,2,3\right),$$

where $g_{i}^{I}(z)$ and $f_{i}^{I}(z)$ (i=1,2,3,...,9) are unknown functions of *z*. After Substituting Equation (32) into Equations (26)–(28), it is found that

(33) $$\left\{ \begin{array}{l} g_{1}^{I}(z)=B_{1}^{I}+B_{2}^{I}e^{-\frac{\alpha }{h}z}-\frac{\alpha }{2h\lambda_{33}^{0}}C_{4}^{0}ze^{-\frac{\alpha }{h}z}\\ g_{2}^{I}(z)=B_{3}^{I}+B_{4}^{I}e^{-\frac{\alpha }{h}z}-\frac{\alpha }{h\lambda_{33}^{0}}C_{8}^{0}ze^{-\frac{\alpha }{h}z}\\ g_{3}^{I}(z)=B_{5}^{I}+B_{6}^{I}e^{-\frac{\alpha }{h}z}-z[\frac{1}{\lambda_{33}^{0}}\frac{h}{\alpha }(\frac{s_{13}^{0}}{s_{11}^{0}}\frac{\alpha }{h}C_{1}^{0}+\frac{s_{44}^{0}}{s_{11}^{0}}C_{2}^{0}+2\lambda_{11}^{0}B_{1}^{I})+\frac{s_{13}^{0}}{2\lambda_{33}^{0}s_{11}^{0}}C_{2}^{0}z]\\ \;+z[\frac{h}{\alpha }\frac{1}{\lambda_{33}^{0}}(-\frac{s_{13}^{0}}{s_{11}^{0}}\frac{\alpha }{h}C_{3}^{0}+\frac{s_{44}^{0}}{s_{11}^{0}}C_{4}^{0}-\frac{\lambda_{11}^{0}}{\lambda_{33}^{0}}C_{4}^{0}-\frac{\alpha^{2}}{h^{2}}C_{12}^{0}+2\lambda_{11}^{0}B_{2}^{I})\\ \;+\frac{1}{2\lambda_{33}^{0}}(\frac{s_{13}^{0}}{s_{11}^{0}}\frac{\alpha }{h}C_{3}^{0}-\frac{s_{13}^{0}+s_{44}^{0}}{s_{11}^{0}}C_{4}^{0}-\frac{\lambda_{11}^{0}}{\lambda_{33}^{0}}C_{4}^{0})z+\frac{s_{13}^{0}}{6s_{11}^{0}\lambda_{33}^{0}}\frac{\alpha }{h}C_{4}^{0}z^{2}]e^{-\frac{\alpha }{h}z} \end{array},\right.$$

(34) $$\left\{ \begin{array}{l} g_{4}^{I}(z)=B_{7}^{I}+B_{8}^{I}e^{-\frac{\alpha }{h}z}\\ g_{5}^{I}(z)=B_{9}^{I}+B_{10}^{I}e^{-\frac{\alpha }{h}z}\\ g_{6}^{I}(z)=B_{11}^{I}+B_{12}^{I}e^{-\frac{\alpha }{h}z}+\frac{1}{\lambda_{33}^{0}}\frac{h}{\alpha }[(\frac{\alpha }{h}C_{1}^{0}-2\lambda_{11}^{0}B_{7}^{I})z+\frac{1}{2}\frac{\alpha }{h}C_{2}^{0}z^{2}+(2\lambda_{11}^{0}B_{8}^{I}-C_{4}^{0})ze^{-\frac{\alpha }{h}z}] \end{array}\right.,$$

(35) $$\left\{ \begin{array}{l} g_{7}^{I}(z)=B_{13}^{I}+B_{14}^{I}e^{-\frac{\alpha }{h}z}\\ g_{8}^{I}(z)=B_{15}^{I}+B_{16}^{I}e^{-\frac{\alpha }{h}z}\\ g_{9}^{I}(z)=B_{17}^{I}+B_{18}^{I}e^{-\frac{\alpha }{h}z}-\frac{1}{\lambda_{33}^{0}}\frac{h}{\alpha }(C_{2}^{0}+2\lambda_{11}^{0}B_{13}^{I})z+\frac{1}{2\lambda_{33}^{0}}(4\frac{h}{\alpha }\lambda_{11}^{0}B_{14}^{I}-2C_{3}^{0}-C_{4}^{0}z)ze^{-\frac{\alpha }{h}z} \end{array}\right.,$$

(36) $$\left\{ \begin{array}{l} f_{3i-2}^{I}(z)=C_{12i-11}^{I}+C_{12i-10}^{I}z+(C_{12i-9}^{I}+C_{12i-8}^{I}z)e^{-\frac{\alpha }{h}z}\\ f_{3i-1}^{I}(z)=C_{12i-7}^{I}+C_{12i-6}^{I}z+(C_{12i-5}^{I}+C_{12i-4}^{I}z)e^{-\frac{\alpha }{h}z} \end{array}\right.\left(i=1,2,3\right)$$

and

(37) $$\left\{ \begin{array}{l} f_{3}^{I}(z)=C_{9}^{I}+C_{10}^{I}z+(C_{11}^{I}+C_{12}^{I}z)e^{-\frac{\alpha }{h}z}-\frac{z^{2}}{6s_{11}^{0}}(3s_{13}^{0}C_{1}^{I}+3s_{44}^{0}\frac{h}{\alpha }C_{2}^{I}+6\frac{h}{\alpha }\frac{\lambda_{11}^{0}}{\lambda_{33}^{0}}B_{1}^{0}+s_{13}^{0}C_{2}^{I}z)\\ \;-\frac{z^{2}}{6s_{11}^{0}}(3s_{13}^{0}C_{3}^{I}-3s_{44}^{0}\frac{h}{\alpha }C_{4}^{I}+s_{13}^{0}C_{4}^{I}z)e^{-\frac{\alpha }{h}z}\\ f_{6}^{I}(z)=C_{21}^{I}+C_{22}^{I}z+(C_{23}^{I}+C_{24}^{I}z)e^{-\frac{\alpha }{h}z}-\frac{z^{2}}{6s_{11}^{0}}(3s_{13}^{0}C_{13}^{I}+3s_{44}^{0}\frac{h}{\alpha }C_{14}^{I}+s_{13}^{0}C_{14}^{I}z)\\ \;-\frac{z^{2}}{6s_{11}^{0}}(3s_{13}^{0}C_{15}^{I}-3s_{44}^{0}\frac{h}{\alpha }C_{16}^{I}+6\frac{h}{\alpha }B_{2}^{0}+s_{13}^{0}C_{16}^{I}z)e^{-\frac{\alpha }{h}z}\\ f_{9}^{I}(z)=C_{33}^{I}+C_{34}^{I}z+(C_{35}^{I}+C_{36}^{I}z)e^{-\frac{\alpha }{h}z}-\frac{z^{2}}{6s_{11}^{0}}(3s_{13}^{0}C_{25}^{I}+3s_{44}^{0}\frac{h}{\alpha }C_{25}^{I}+6\frac{h}{\alpha }B_{1}^{0}+s_{13}^{0}C_{26}^{I}z)\\ \;-\frac{z^{2}}{6s_{11}^{0}}(3s_{13}^{0}C_{27}^{I}-3s_{44}^{0}\frac{h}{\alpha }C_{28}^{I}+s_{13}^{0}C_{28}^{I}z)e^{-\frac{\alpha }{h}z} \end{array},\right.$$

where $B_{i}^{I}$(i=1,2,3,...,18) and $C_{i}^{I}$(i=1,2,3,...,36) are undetermined constants which can be determined by Equation (29)–(31), please see Appendix A.

3. Similarly, by comparing the coefficients of ${\left(d_{31}^{0}\right)}^{2}$, ${\left(d_{33}^{0}\right)}^{2}$, ${\left(d_{15}^{0}\right)}^{2}$, $d_{31}^{0}d_{33}^{0}$, $d_{31}^{0}d_{15}^{0}$, and $d_{33}^{0}d_{15}^{0}$ in Equation (16), we may obtain the differential equations for $\Phi_{i}^{\mathrm{II}}$ and $U_{i}^{\mathrm{II}}$ (i=1,2,3,...,6), respectively, for term ${\left(d_{31}^{0}\right)}^{2}$:

(38) $$\left\{ \begin{array}{l} \lambda_{33}^{0}\frac{\alpha }{h}\frac{\partial \Phi_{1}^{\mathrm{II}}}{\partial z}+\lambda_{33}^{0}\frac{\partial^{2}\Phi_{1}^{\mathrm{II}}}{\partial z^{2}}+\lambda_{11}^{0}\frac{\partial^{2}\Phi_{1}^{\mathrm{II}}}{\partial x^{2}}=\frac{\alpha }{h}\frac{\partial^{2}U_{1}^{I}}{\partial z^{2}}+\frac{\partial^{3}U_{1}^{I}}{\partial z^{3}}\\ s_{11}^{0}\frac{\alpha^{2}}{h^{2}}\frac{\partial^{2}U_{1}^{\mathrm{II}}}{\partial z^{2}}+s_{13}^{0}\frac{\alpha^{2}}{h^{2}}\frac{\partial^{2}U_{1}^{\mathrm{II}}}{\partial x^{2}}+2s_{11}^{0}\frac{\alpha }{h}\frac{\partial^{3}U_{1}^{\mathrm{II}}}{\partial z^{3}}+(2s_{13}^{0}+s_{44}^{0})\frac{\alpha }{h}\frac{\partial^{3}U_{1}^{\mathrm{II}}}{\partial x^{2}\partial z}+s_{11}^{0}\frac{\partial^{4}U_{1}^{\mathrm{II}}}{\partial z^{4}}\\ +s_{33}^{0}\frac{\partial^{4}U_{1}^{\mathrm{II}}}{\partial x^{4}}+(s_{44}^{0}+2s_{13}^{0})\frac{\partial^{4}U_{1}^{\mathrm{II}}}{\partial x^{2}\partial z^{2}}=\frac{\alpha^{2}}{h^{2}}\frac{\partial \Phi_{1}^{I}}{\partial z}-2\frac{\alpha }{h}\frac{\partial^{2}\Phi_{1}^{I}}{\partial z^{2}}+\frac{\partial^{3}\Phi_{1}^{I}}{\partial z^{3}} \end{array},\right.$$

for term ${\left(d_{33}^{0}\right)}^{2}$:

(39) $$\left\{ \begin{array}{l} \lambda_{33}^{0}\frac{\alpha }{h}\frac{\partial \Phi_{2}^{\mathrm{II}}}{\partial z}+\lambda_{33}^{0}\frac{\partial^{2}\Phi_{2}^{\mathrm{II}}}{\partial z^{2}}+\lambda_{11}^{0}\frac{\partial^{2}\Phi_{2}^{\mathrm{II}}}{\partial x^{2}}=\frac{\alpha }{h}\frac{\partial^{2}U_{2}^{I}}{\partial x^{2}}+\frac{\partial^{3}U_{2}^{I}}{\partial x^{2}\partial z}\\ s_{11}^{0}\frac{\alpha^{2}}{h^{2}}\frac{\partial^{2}U_{2}^{\mathrm{II}}}{\partial z^{2}}+s_{13}^{0}\frac{\alpha^{2}}{h^{2}}\frac{\partial^{2}U_{2}^{\mathrm{II}}}{\partial x^{2}}+2s_{11}^{0}\frac{\alpha }{h}\frac{\partial^{3}U_{2}^{\mathrm{II}}}{\partial z^{3}}+(2s_{13}^{0}+s_{44}^{0})\frac{\alpha }{h}\frac{\partial^{3}U_{2}^{\mathrm{II}}}{\partial x^{2}\partial z}\\ +s_{11}^{0}\frac{\partial^{4}U_{2}^{\mathrm{II}}}{\partial z^{4}}+s_{33}^{0}\frac{\partial^{4}U_{2}^{\mathrm{II}}}{\partial x^{4}}+(s_{44}^{0}+2s_{13}^{0})\frac{\partial^{4}U_{2}^{\mathrm{II}}}{\partial x^{2}\partial z^{2}}=\frac{\partial^{3}\Phi_{2}^{I}}{\partial x^{2}\partial z} \end{array},\right.$$

for term ${\left(d_{15}^{0}\right)}^{2}$:

(40) $$\left\{ \begin{array}{l} \lambda_{33}^{0}\frac{\alpha }{h}\frac{\partial \Phi_{3}^{\mathrm{II}}}{\partial z}+\lambda_{33}^{0}\frac{\partial^{2}\Phi_{3}^{\mathrm{II}}}{\partial z^{2}}+\lambda_{11}^{0}\frac{\partial^{2}\Phi_{3}^{\mathrm{II}}}{\partial x^{2}}=-\frac{\partial^{3}U_{3}^{I}}{\partial x^{2}\partial z}\\ s_{11}^{0}\frac{\alpha^{2}}{h^{2}}\frac{\partial^{2}U_{3}^{\mathrm{II}}}{\partial z^{2}}+s_{13}^{0}\frac{\alpha^{2}}{h^{2}}\frac{\partial^{2}U_{3}^{\mathrm{II}}}{\partial x^{2}}+2s_{11}^{0}\frac{\alpha }{h}\frac{\partial^{3}U_{3}^{\mathrm{II}}}{\partial z^{3}}+(2s_{13}^{0}+s_{44}^{0})\frac{\alpha }{h}\frac{\partial^{3}U_{3}^{\mathrm{II}}}{\partial x^{2}\partial z}+s_{11}^{0}\frac{\partial^{4}U_{3}^{\mathrm{II}}}{\partial z^{4}}\\ +s_{33}^{0}\frac{\partial^{4}U_{3}^{\mathrm{II}}}{\partial x^{4}}+(s_{44}^{0}+2s_{13}^{0})\frac{\partial^{4}U_{3}^{\mathrm{II}}}{\partial x^{2}\partial z^{2}}=-\frac{\partial^{3}\Phi_{3}^{I}}{\partial x^{2}\partial z}-\frac{\alpha }{h}\frac{\partial^{2}\Phi_{3}^{I}}{\partial x^{2}} \end{array},\right.$$

for term $d_{31}^{0}d_{33}^{0}$:

(41) $$\left\{ \begin{array}{l} \lambda_{33}^{0}\frac{\alpha }{h}\frac{\partial \Phi_{4}^{\mathrm{II}}}{\partial z}+\lambda_{33}^{0}\frac{\partial^{2}\Phi_{4}^{\mathrm{II}}}{\partial z^{2}}+\lambda_{11}^{0}\frac{\partial^{2}\Phi_{4}^{\mathrm{II}}}{\partial x^{2}}=\frac{\alpha }{h}\frac{\partial^{2}U_{2}^{I}}{\partial z^{2}}+\frac{\partial^{3}U_{2}^{I}}{\partial z^{3}}+\frac{\alpha }{h}\frac{\partial^{2}U_{1}^{I}}{\partial x^{2}}+\frac{\partial^{3}U_{1}^{I}}{\partial x^{2}\partial z}\\ s_{11}^{0}\frac{\alpha^{2}}{h^{2}}\frac{\partial^{2}U_{4}^{\mathrm{II}}}{\partial z^{2}}+s_{13}^{0}\frac{\alpha^{2}}{h^{2}}\frac{\partial^{2}U_{4}^{\mathrm{II}}}{\partial x^{2}}+2s_{11}^{0}\frac{\alpha }{h}\frac{\partial^{3}U_{4}^{\mathrm{II}}}{\partial z^{3}}+(2s_{13}^{0}+s_{44}^{0})\frac{\alpha }{h}\frac{\partial^{3}U_{4}^{\mathrm{II}}}{\partial x^{2}\partial z}+s_{11}^{0}\frac{\partial^{4}U_{4}^{\mathrm{II}}}{\partial z^{4}}\\ +s_{33}^{0}\frac{\partial^{4}U_{4}^{\mathrm{II}}}{\partial x^{4}}+(s_{44}^{0}+2s_{13}^{0})\frac{\partial^{4}U_{4}^{\mathrm{II}}}{\partial x^{2}\partial z^{2}}=\frac{\alpha^{2}}{h^{2}}\frac{\partial \Phi_{2}^{I}}{\partial z}+2\frac{\alpha }{h}\frac{\partial^{2}\Phi_{2}^{I}}{\partial z^{2}}+\frac{\partial^{3}\Phi_{2}^{I}}{\partial z^{3}}+\frac{\partial^{3}\Phi_{1}^{I}}{\partial x^{2}\partial z} \end{array},\right.$$

for term $d_{31}^{0}d_{15}^{0}$:

(42) $$\left\{ \begin{array}{l} \lambda_{33}^{0}\frac{\alpha }{h}\frac{\partial \Phi_{5}^{\mathrm{II}}}{\partial z}+\lambda_{33}^{0}\frac{\partial^{2}\Phi_{5}^{\mathrm{II}}}{\partial z^{2}}+\lambda_{11}^{0}\frac{\partial^{2}\Phi_{5}^{\mathrm{II}}}{\partial x^{2}}=\frac{\alpha }{h}\frac{\partial^{2}U_{3}^{I}}{\partial z^{2}}+\frac{\partial^{3}U_{3}^{I}}{\partial z^{3}}-\frac{\partial^{3}U_{1}^{I}}{\partial x^{2}\partial z}\\ s_{11}^{0}\frac{\alpha^{2}}{h^{2}}\frac{\partial^{2}U_{5}^{\mathrm{II}}}{\partial z^{2}}+s_{13}^{0}\frac{\alpha^{2}}{h^{2}}\frac{\partial^{2}U_{5}^{\mathrm{II}}}{\partial x^{2}}+2s_{11}^{0}\frac{\alpha }{h}\frac{\partial^{3}U_{5}^{\mathrm{II}}}{\partial z^{3}}+(2s_{13}^{0}+s_{44}^{0})\frac{\alpha }{h}\frac{\partial^{3}U_{5}^{\mathrm{II}}}{\partial x^{2}\partial z}+s_{11}^{0}\frac{\partial^{4}U_{5}^{\mathrm{II}}}{\partial z^{4}}\\ +s_{33}^{0}\frac{\partial^{4}U_{5}^{\mathrm{II}}}{\partial x^{4}}+(s_{44}^{0}+2s_{13}^{0})\frac{\partial^{4}U_{5}^{\mathrm{II}}}{\partial x^{2}\partial z^{2}}=\frac{\alpha^{2}}{h^{2}}\frac{\partial \Phi_{3}^{I}}{\partial z}+2\frac{\alpha }{h}\frac{\partial^{2}\Phi_{3}^{I}}{\partial z^{2}}+\frac{\partial^{3}\Phi_{3}^{I}}{\partial z^{3}}-\frac{\partial^{3}\Phi_{1}^{I}}{\partial x^{2}\partial z}-\frac{\alpha }{h}\frac{\partial^{2}\Phi_{1}^{I}}{\partial x^{2}} \end{array},\right.$$

and for term $d_{33}^{0}d_{15}^{0}$:

(43) $$\left\{ \begin{array}{l} \lambda_{33}^{0}\frac{\alpha }{h}\frac{\partial \Phi_{6}^{\mathrm{II}}}{\partial z}+\lambda_{33}^{0}\frac{\partial^{2}\Phi_{6}^{\mathrm{II}}}{\partial z^{2}}+\lambda_{11}^{0}\frac{\partial^{2}\Phi_{6}^{\mathrm{II}}}{\partial x^{2}}=\frac{\alpha }{h}\frac{\partial^{2}U_{3}^{I}}{\partial x^{2}}+\frac{\partial^{3}U_{3}^{I}}{\partial x^{2}\partial z}-\frac{\partial^{3}U_{2}^{I}}{\partial x^{2}\partial z}\\ s_{11}^{0}\frac{\alpha^{2}}{h^{2}}\frac{\partial^{2}U_{6}^{\mathrm{II}}}{\partial z^{2}}+s_{13}^{0}\frac{\alpha^{2}}{h^{2}}\frac{\partial^{2}U_{6}^{\mathrm{II}}}{\partial x^{2}}+2s_{11}^{0}\frac{\alpha }{h}\frac{\partial^{3}U_{6}^{\mathrm{II}}}{\partial z^{3}}+(2s_{13}^{0}+s_{44}^{0})\frac{\alpha }{h}\frac{\partial^{3}U_{6}^{\mathrm{II}}}{\partial x^{2}\partial z}+s_{11}^{0}\frac{\partial^{4}U_{6}^{\mathrm{II}}}{\partial z^{4}}\\ +s_{33}^{0}\frac{\partial^{4}U_{6}^{\mathrm{II}}}{\partial x^{4}}+(s_{44}^{0}+2s_{13}^{0})\frac{\partial^{4}U_{6}^{\mathrm{II}}}{\partial x^{2}\partial z^{2}}=\frac{\partial^{3}\Phi_{3}^{I}}{\partial x^{2}\partial z}-\frac{\partial^{3}\Phi_{2}^{I}}{\partial x^{2}\partial z}-\frac{\alpha }{h}\frac{\partial^{2}\Phi_{2}^{I}}{\partial x^{2}} \end{array},\right.$$

which may be solved under the boundary conditions

(44) $$\int_{-h/2}^{h/2}\left(-\frac{\partial^{2}U_{i}^{\mathrm{II}}}{\partial z\partial x}dz\right)=0,\int_{-h/2}^{h/2}\frac{\partial^{2}U_{i}^{\mathrm{II}}}{\partial z^{2}}dz=0\;\mathrm{and}\;\int_{-h/2}^{h/2}z\frac{\partial^{2}U_{i}^{\mathrm{II}}}{\partial z^{2}}dz=0\;\left(i=1,2,3,...,6\right),\;\mathrm{at}\;x=0,$$

(45) $$\left\{ \begin{array}{l} \frac{\partial^{2}U_{i}^{\mathrm{II}}}{\partial x^{2}}=-\frac{\partial^{2}U_{i}^{\mathrm{II}}}{\partial z\partial x}=0,\;\mathrm{at}\;z=h/2\\ \frac{\partial^{2}U_{i}^{\mathrm{II}}}{\partial x^{2}}=-\frac{\partial^{2}U_{i}^{\mathrm{II}}}{\partial z\partial x}=0,\;\mathrm{at}\;z=-h/2 \end{array}\right.\;\left(i=1,2,3,...,6\right)$$

and

(46) $$\left\{ \begin{array}{l} \int_{-h/2}^{h/2}(-d_{15}\frac{\partial^{2}U_{i}^{\mathrm{II}}}{\partial z\partial x}-\lambda_{11}\frac{\partial \Phi_{i}^{\mathrm{II}}}{\partial x})dz=0,\;\mathrm{at}x=0\;\mathrm{and}\;x=l\\ d_{31}\frac{\partial^{2}U_{i}^{\mathrm{II}}}{\partial z^{2}}+d_{33}\frac{\partial^{2}U_{i}^{\mathrm{II}}}{\partial x^{2}}-\lambda_{33}\frac{\partial \Phi_{i}^{\mathrm{II}}}{\partial z}=0,\;\mathrm{at}z=h/2\;\mathrm{and}\;z=-h/2 \end{array}\right.\;\left(i=1,2,3,...,6\right).$$

Suppose

(47) $$\left\{ \begin{array}{l} \Phi_{i}^{\mathrm{II}}=x^{2}g_{3i-2}^{\mathrm{II}}(z)+xg_{3i-1}^{\mathrm{II}}(z)+g_{3i}^{\mathrm{II}}(z)\\ U_{i}^{\mathrm{II}}=\frac{x^{2}}{2}f_{3i-2}^{\mathrm{II}}(z)+xf_{3i-1}^{\mathrm{II}}(z)+f_{3i}^{\mathrm{II}}(z) \end{array}\right.\left(i=1,2,3,...,6\right)$$

where $g_{i}^{\mathrm{II}}(z)$ and $f_{i}^{\mathrm{II}}(z)$ (i=1,2,3,...,18) are unknown functions of *z*. After Substituting Equation (47) into Equations (38)–(43), it is found that

(48) $$\left\{ \begin{array}{l} g_{3i-2}^{\mathrm{II}}(z)=B_{6i-5}^{\mathrm{II}}+B_{6i-4}^{\mathrm{II}}e^{-\frac{\alpha }{h}z}\\ g_{3i-1}^{\mathrm{II}}(z)=B_{6i-3}^{\mathrm{II}}+B_{6i-2}^{\mathrm{II}}e^{-\frac{\alpha }{h}z}\\ g_{3i}^{\mathrm{II}}(z)=B_{6i-1}^{\mathrm{II}}+B_{6i}^{\mathrm{II}}e^{-\frac{\alpha }{h}z}-2\frac{h}{\alpha }\frac{\lambda_{11}^{0}}{\lambda_{33}^{0}}B_{6i-5}^{\mathrm{II}}z+2\frac{h}{\alpha }\frac{\lambda_{11}^{0}}{\lambda_{33}^{0}}B_{6i-4}^{\mathrm{II}}ze^{-\frac{\alpha }{h}z} \end{array}\right.\;\left(i=1,2,3,...,6\right),$$

(49) $$\left\{ \begin{array}{l} f_{1}^{\mathrm{II}}=C_{1}^{\mathrm{II}}+2\frac{1}{s_{11}^{0}}B_{1}^{I}z+C_{2}^{\mathrm{II}}z-\frac{h}{\alpha }[2\frac{1}{s_{11}^{0}}B_{2}^{I}+C_{3}^{\mathrm{II}}-\frac{C_{4}^{0}}{s_{11}^{0}\lambda_{33}^{0}}(\frac{\alpha }{h}z+1)+C_{4}^{\mathrm{II}}\frac{h}{\alpha }(\frac{\alpha }{h}z+1)]e^{-\frac{\alpha }{h}z}\\ f_{2}^{\mathrm{II}}=C_{5}^{\mathrm{II}}+\frac{1}{s_{11}^{0}}B_{3}^{I}z+C_{6}^{\mathrm{II}}z-\frac{h}{\alpha }[\frac{1}{s_{11}^{0}}B_{4}^{I}+C_{7}^{\mathrm{II}}-\frac{C_{8}^{0}}{s_{11}^{0}\lambda_{11}^{0}}(\frac{\alpha }{h}z+1)+C_{8}^{\mathrm{II}}\frac{h}{\alpha }(\frac{\alpha }{h}z+1)]e^{-\frac{\alpha }{h}z}\\ f_{3}^{\mathrm{II}}=C_{9}^{\mathrm{II}}+C_{10}^{\mathrm{II}}z+\frac{1}{s_{11}^{0}}B_{5}^{I}z-\frac{1}{2}(2\frac{h}{\alpha }\frac{1}{s_{11}^{0}}\frac{s_{44}^{0}}{s_{11}^{0}}B_{1}^{I}+\frac{s_{13}^{0}}{s_{11}^{0}}C_{1}^{\mathrm{II}}+\frac{h}{\alpha }\frac{s_{44}^{0}}{s_{11}^{0}}C_{2}^{\mathrm{II}})z^{2}\\ \;-\frac{1}{2s_{11}^{0}}\frac{1}{\lambda_{33}^{0}s_{11}^{0}}\frac{h}{\alpha }(s_{13}^{0}\frac{\alpha }{h}C_{1}^{0}+s_{44}^{0}C_{2}^{0}+2\lambda_{11}^{0}s_{11}^{0}B_{1}^{I})z^{2}-\frac{s_{13}^{0}}{3s_{11}^{0}}(\frac{1}{s_{11}^{0}}B_{1}^{I}+\frac{1}{2}C_{2}^{\mathrm{II}})z^{3}\\ \;-\frac{1}{6s_{11}^{0}}\frac{s_{13}^{0}}{\lambda_{33}^{0}s_{11}^{0}}C_{2}^{0}z^{3}-(\frac{h}{\alpha }F_{1}+\frac{h^{2}}{\alpha^{2}}G_{1}+2\frac{h^{3}}{\alpha^{3}}H_{1}+6\frac{h^{4}}{\alpha^{4}}I_{1}+\frac{h}{\alpha }G_{1}z+2\frac{h^{2}}{\alpha^{2}}H_{1}z\\ \;+6\frac{h^{3}}{\alpha^{3}}I_{1}z+\frac{h}{\alpha }H_{1}z^{2}+3\frac{h^{2}}{\alpha^{2}}I_{1}z^{2}+\frac{h}{\alpha }I_{1}z^{3})e^{-\frac{\alpha }{h}z} \end{array},\right.$$

(50) $$\left\{ \begin{array}{l} f_{3i-2}^{\mathrm{II}}=C_{12i-11}^{\mathrm{II}}+C_{12i-10}^{\mathrm{II}}z+(C_{12i-9}^{\mathrm{II}}+C_{12i-8}^{\mathrm{II}}z)e^{-\frac{\alpha }{h}z}\\ f_{3i-1}^{\mathrm{II}}=C_{12i-7}^{\mathrm{II}}+C_{12i-6}^{\mathrm{II}}z+(C_{12i-5}^{\mathrm{II}}+C_{12i-4}^{\mathrm{II}}z)e^{-\frac{\alpha }{h}z} \end{array}\right.\;\;(i=2,3,...,6)$$

and

(51) $$\left\{ \begin{array}{l} f_{6}^{\mathrm{II}}=C_{21}^{\mathrm{II}}+C_{22}^{\mathrm{II}}z+(C_{23}^{\mathrm{II}}+C_{24}^{\mathrm{II}}z)e^{-\frac{\alpha }{h}z}-\frac{z^{2}}{6s_{11}^{0}}(3s_{13}^{0}C_{13}^{\mathrm{II}}+3s_{44}^{0}\frac{h}{\alpha }C_{14}^{\mathrm{II}}+s_{13}^{0}C_{14}^{\mathrm{II}}z)\\ \;-\frac{z^{2}}{6s_{11}^{0}}(3s_{13}^{0}C_{15}^{\mathrm{II}}-3s_{44}^{0}\frac{h}{\alpha }C_{16}^{\mathrm{II}}+s_{13}^{0}C_{16}^{\mathrm{II}}z)e^{-\frac{\alpha }{h}z}\\ f_{9}^{\mathrm{II}}=C_{33}^{\mathrm{II}}+C_{34}^{\mathrm{II}}z+(C_{35}^{\mathrm{II}}+C_{36}^{\mathrm{II}}z)e^{-\frac{\alpha }{h}z}-\frac{z^{2}}{6s_{11}^{0}}(3s_{13}^{0}C_{25}^{\mathrm{II}}+3s_{44}^{0}\frac{h}{\alpha }C_{26}^{\mathrm{II}}+6\frac{\alpha }{h}B_{13}^{I}+s_{13}^{0}C_{26}^{\mathrm{II}}z)\\ \;-\frac{z^{2}}{6s_{11}^{0}}(3s_{13}^{0}C_{27}^{\mathrm{II}}-3s_{44}^{0}\frac{h}{\alpha }C_{28}^{\mathrm{II}}+s_{13}^{0}C_{28}^{\mathrm{II}}z)e^{-\frac{\alpha }{h}z}\\ f_{12}^{\mathrm{II}}=C_{45}^{\mathrm{II}}+C_{46}^{\mathrm{II}}z+\frac{1}{s_{11}^{0}}B_{11}^{I}z-\frac{1}{2}(\frac{s_{13}^{0}}{s_{11}^{0}}C_{37}^{\mathrm{II}}+\frac{h}{\alpha }\frac{s_{44}^{0}}{s_{11}^{0}}C_{38}^{\mathrm{II}}-\frac{1}{\lambda_{33}^{0}}\frac{1}{s_{11}^{0}}C_{1}^{0})z^{2}+\frac{1}{6}(\frac{1}{\lambda_{33}^{0}}\frac{1}{s_{11}^{0}}C_{2}^{0}-\frac{s_{13}^{0}}{s_{11}^{0}}C_{38}^{\mathrm{II}})z^{3}-(\frac{h}{\alpha }F_{2}\\ \;+\frac{h^{2}}{\alpha^{2}}G_{2}+2\frac{h^{3}}{\alpha^{3}}H_{2}+6\frac{h^{4}}{\alpha^{4}}I_{2}+\frac{h}{\alpha }G_{2}z+2\frac{h^{2}}{\alpha^{2}}H_{2}z+6\frac{h^{3}}{\alpha^{3}}I_{2}z+\frac{h}{\alpha }H_{2}z^{2}+3\frac{h^{2}}{\alpha^{2}}I_{2}z^{2}+\frac{h}{\alpha }I_{2}z^{3})e^{-\frac{\alpha }{h}z}\\ f_{15}^{\mathrm{II}}=\frac{1}{s_{11}^{0}}[C_{57}^{\mathrm{II}}+s_{11}^{0}C_{58}^{\mathrm{II}}z+B_{17}^{I}z-\frac{h}{\alpha }B_{1}^{I}z^{2}-\frac{1}{2\lambda_{33}^{0}}\frac{h}{\alpha }(C_{2}^{0}+2\lambda_{11}^{0}B_{13}^{I})z^{2}\\ \;-(\frac{h}{\alpha }F_{3}+\frac{h^{2}}{\alpha^{2}}G_{3}+2\frac{h^{3}}{\alpha^{3}}H_{3}+\frac{h}{\alpha }G_{3}z+2\frac{h^{2}}{\alpha^{2}}H_{3}z+\frac{h}{\alpha }H_{3}z^{2})e^{-\frac{\alpha }{h}z}]\\ f_{18}^{\mathrm{II}}=C_{69}^{\mathrm{II}}+C_{70}^{\mathrm{II}}z+(C_{71}^{\mathrm{II}}+C_{72}^{\mathrm{II}}z)e^{-\frac{\alpha }{h}z}-\frac{z^{2}}{6s_{11}^{0}}(3s_{13}^{0}C_{61}^{\mathrm{II}}+3s_{44}^{0}\frac{h}{\alpha }C_{62}^{\mathrm{II}}+s_{13}^{0}C_{62}^{\mathrm{II}}z)\\ \;-\frac{z^{2}}{6s_{11}^{0}}(3s_{13}^{0}C_{63}^{\mathrm{II}}-3s_{44}^{0}\frac{h}{\alpha }C_{64}^{\mathrm{II}}+s_{13}^{0}C_{64}^{\mathrm{II}}z)e^{-\frac{\alpha }{h}z} \end{array},\right.$$

where *F_i_* (i=1,2,3), *G_i_* (i=1,2,3), *H_i_* (i=1,2,3), and *I_i_* (i=1,2) can be found in Appendix A, and $B_{i}^{\mathrm{II}}$ (i=1,2,3,...,36) and $C_{i}^{\mathrm{II}}$ (i=1,2,3,...,72) are undetermined constants which can be determined by Equations (44)–(46) , please see Appendix A. 

Thus, the expression of the electric potential function Φ(*x*, *z*) and Airy stress function *U*(*x*, *z*) may be obtained by means of Equations (17) and (18), Equations (23)–(25), Equations (32)–(37), and Equations (47)–(51). Substituting Equations (17) and (18) into Equations (7) and (8), the electric field components and the stress components may be expressed as

(52) $$\left\{ \begin{array}{l} E_{x}=-(2xg_{1}^{I}+g_{2}^{I})d_{31}^{0}-(2xg_{7}^{I}+g_{8}^{I})d_{15}^{0}\\ E_{z}=-(x^{2}{g_{1}^{I}}^{\prime }+x{g_{2}^{I}}^{\prime }+{g_{3}^{I}}^{\prime })d_{31}^{0}-{g_{6}^{I}}^{\prime }d_{33}^{0}-{g_{9}^{I}}^{\prime }d_{15}^{0} \end{array}\right.$$

and

(53) $$\left\{ \begin{array}{l} \sigma_{x}=\frac{x^{2}}{2}{f_{1}^{0}}^{\prime \prime }+x{f_{2}^{0}}^{\prime \prime }+{f_{3}^{0}}^{\prime \prime }+{f_{3}^{II}}^{\prime \prime }{\left(d_{31}^{0}\right)}^{2}+{f_{9}^{II}}^{\prime \prime }{\left(d_{15}^{0}\right)}^{2}+{f_{12}^{II}}^{\prime \prime }d_{31}^{0}d_{33}^{0}+{f_{15}^{II}}^{\prime \prime }d_{31}^{0}d_{15}^{0}\\ \sigma_{z}=f_{1}^{0}\\ \tau_{zx}=-x{f_{1}^{0}}^{\prime }-{f_{2}^{0}}^{\prime } \end{array}\right..$$

And substituting Equations (52) and (53) into Equation (4), the electric displacement components and the strain components may be written as

(54) $$\left\{ \begin{array}{l} D_{x}=-[\lambda_{11}^{0}(2xg_{1}^{I}+g_{2}^{I})d_{31}^{0}+\lambda_{11}^{0}(2xg_{7}^{I}+g_{8}^{I})d_{15}^{0}+(x{f_{1}^{0}}^{\prime }+{f_{2}^{0}}^{\prime })d_{15}^{0}]e^{\alpha z/h}\\ D_{z}=(\frac{x^{2}}{2}{f_{1}^{0}}^{\prime \prime }+x{f_{2}^{0}}^{\prime \prime }+{f_{3}^{0}}^{\prime \prime })d_{31}^{0}e^{\alpha z/h}+f_{1}^{0}d_{33}^{0}e^{\alpha z/h}\\ \;\;-[(x^{2}{g_{1}^{I}}^{\prime }+x{g_{2}^{I}}^{\prime }+{g_{3}^{I}}^{\prime })d_{31}^{0}+{g_{6}^{I}}^{\prime }d_{33}^{0}+{g_{9}^{I}}^{\prime }d_{15}^{0}]\lambda_{33}^{0}e^{\alpha z/h} \end{array}\right.$$

and

(55) $$\left\{ \begin{array}{l} \varepsilon_{x}=[(s_{11}^{0}\frac{x^{2}}{2}{f_{1}^{0}}^{\prime \prime }+s_{11}^{0}x{f_{2}^{0}}^{\prime \prime }+s_{11}^{0}{f_{3}^{0}}^{\prime \prime }+s_{13}^{0}f_{1}^{I})+(s_{11}^{0}{f_{3}^{\mathrm{II}}}^{\prime \prime }-x^{2}{g_{1}^{I}}^{\prime }-x{g_{2}^{I}}^{\prime }-{g_{3}^{I}}^{\prime }){\left(d_{31}^{0}\right)}^{2}\\ \;+s_{11}^{0}{f_{9}^{\mathrm{II}}}^{\prime \prime }{\left(d_{15}^{0}\right)}^{2}+(s_{11}^{0}{f_{12}^{\mathrm{II}}}^{\prime \prime }-{g_{6}^{I}}^{\prime })d_{31}^{0}d_{33}^{0}+(s_{11}^{0}{f_{15}^{\mathrm{II}}}^{\prime \prime }-{g_{9}^{I}}^{\prime })d_{31}^{0}d_{15}^{0}]e^{\alpha z/h}\\ \varepsilon_{z}=[(s_{13}^{0}\frac{x^{2}}{2}{f_{1}^{0}}^{\prime \prime }+s_{13}^{0}x{f_{2}^{0}}^{\prime \prime }+s_{13}^{0}{f_{3}^{0}}^{\prime \prime }+s_{33}^{0}f_{1}^{0})+s_{13}^{0}{f_{3}^{\mathrm{II}}}^{\prime \prime }{\left(d_{31}^{0}\right)}^{2}-{g_{6}^{I}}^{\prime }{\left(d_{33}^{0}\right)}^{2}+s_{13}^{0}{f_{9}^{\mathrm{II}}}^{\prime \prime }{\left(d_{15}^{0}\right)}^{2}\\ \;+(s_{13}^{0}{f_{12}^{\mathrm{II}}}^{\prime \prime }-x^{2}{g_{1}^{I}}^{\prime }-x{g_{2}^{I}}^{\prime }-{g_{3}^{I}}^{\prime })d_{31}^{0}d_{33}^{0}+s_{13}^{0}{f_{15}^{\mathrm{II}}}^{\prime \prime }d_{31}^{0}d_{15}^{0}-{g_{9}^{I}}^{\prime }d_{33}^{0}d_{15}^{0}]e^{\alpha z/h}\\ \gamma_{zx}=-[(x{f_{1}^{0}}^{\prime }+{f_{2}^{0}}^{\prime })s_{44}^{0}+(2xg_{7}^{I}+g_{8}^{I}){\left(d_{15}^{0}\right)}^{2}+(2xg_{1}^{I}+g_{2}^{I})d_{31}^{0}d_{15}^{0}]e^{\alpha z/h} \end{array}.\right.$$

Substituting Equation (55) into the first two items of Equation (5), and integrating with respect to *x* and *z*, respectively, the displacement components may be obtained as

(56) $$\begin{array}{c} u=[(\frac{x^{3}}{6}s_{11}^{0}{f_{1}^{0}}^{\prime \prime }+\frac{x^{2}}{2}s_{11}^{0}{f_{2}^{0}}^{\prime \prime }+xs_{11}^{0}{f_{3}^{0}}^{\prime \prime }+xs_{13}^{0}f_{1}^{I})-(\frac{x^{3}}{3}{g_{1}^{I}}^{\prime }+\frac{x^{2}}{2}{g_{2}^{I}}^{\prime }+x{g_{3}^{I}}^{\prime }-xs_{11}^{0}{f_{3}^{\mathrm{II}}}^{\prime \prime }){\left(d_{31}^{0}\right)}^{2}\\ +xs_{11}^{0}{f_{9}^{\mathrm{II}}}^{\prime \prime }{\left(d_{15}^{0}\right)}^{2}-x({g_{6}^{I}}^{\prime }-s_{11}^{0}{f_{12}^{\mathrm{II}}}^{\prime \prime })d_{31}^{0}d_{33}^{0}-x({g_{9}^{I}}^{\prime }-s_{11}^{0}{f_{15}^{\mathrm{II}}}^{\prime \prime })d_{31}^{0}d_{15}^{0}]e^{\alpha z/h}+g_{1}(z) \end{array}$$

and

(57) $$\begin{array}{l} w=s_{13}^{0}[\frac{x^{2}}{2}(-2\frac{\alpha }{h}C_{4}^{0}+\frac{\alpha^{2}}{h^{2}}C_{3}^{0})+x(-2\frac{\alpha }{h}C_{8}^{0}+\frac{\alpha^{2}}{h^{2}}C_{7}^{0})+\frac{s_{33}^{0}}{s_{13}^{0}}C_{3}^{0}-2\frac{\alpha }{h}C_{12}^{0}+\frac{\alpha^{2}}{h^{2}}C_{11}^{0}-\frac{s_{13}^{0}}{s_{11}^{0}}C_{3}^{0}+\frac{s_{44}^{0}}{s_{11}^{0}}\frac{h}{\alpha }C_{4}^{0}]z\\ \;+s_{13}^{0}[\frac{x^{2}}{4}\frac{\alpha^{2}}{h^{2}}C_{4}^{0}+\frac{1}{2}\frac{\alpha^{2}}{h^{2}}C_{8}^{0}x+\frac{1}{2}\frac{s_{33}^{0}}{s_{13}^{0}}C_{4}^{0}+\frac{1}{2}\frac{\alpha^{2}}{h^{2}}C_{12}^{0}-\frac{s_{13}^{0}}{s_{11}^{0}}\frac{\alpha }{h}C_{3}^{0}-\frac{s_{44}^{0}}{s_{11}^{0}}C_{4}^{0}-\frac{1}{2}\frac{s_{13}^{0}}{s_{11}^{0}}C_{4}^{0}]z^{2}-s_{13}^{0}\frac{\alpha }{h}(\frac{s_{13}^{0}}{6s_{11}^{0}}\frac{\alpha }{h}C_{3}^{0}\\ \;-\frac{2s_{13}^{0}+s_{44}^{0}}{6s_{11}^{0}}C_{4}^{0})z^{3}-s_{13}^{0}\frac{s_{13}^{0}}{24s_{11}^{0}}\frac{\alpha^{2}}{h^{2}}C_{4}^{0}z^{4}+s_{13}^{0}\frac{h}{\alpha }[\frac{s_{33}^{0}}{s_{13}^{0}}C_{1}^{0}-\frac{s_{13}^{0}}{s_{11}^{0}}C_{1}^{0}-\frac{h}{\alpha }\frac{s_{33}^{0}}{s_{13}^{0}}C_{2}^{0}+(1-\frac{s_{44}^{0}}{s_{13}^{0}})\frac{h}{\alpha }\frac{s_{13}^{0}}{s_{11}^{0}}C_{2}^{0}+(\frac{s_{33}^{0}}{s_{13}^{0}}\\ \;-\frac{s_{13}^{0}}{s_{11}^{0}})C_{2}^{0}z]e^{\alpha z/h}+[s_{13}^{0}(G_{1}-\frac{\alpha }{h}F_{1})z+s_{13}^{0}(H_{1}-\frac{\alpha }{2h}G_{1})z^{2}+(\frac{\alpha }{h}I_{1}-\frac{\alpha^{2}}{3h^{2}}H_{1})z^{3}-\frac{1}{4}\frac{\alpha }{h}s_{13}^{0}I_{1}z^{4}+\frac{h^{2}}{\alpha^{2}}\frac{s_{13}^{0}}{\lambda_{33}^{0}s_{11}^{0}}\\ \;\times (\frac{s_{13}^{0}}{s_{11}^{0}}C_{2}^{0}-\frac{s_{13}^{0}}{s_{11}^{0}}\frac{\alpha }{h}C_{1}^{0}+\frac{s_{44}^{0}}{s_{11}^{0}}C_{2}^{0}+2\lambda_{11}^{0}B_{1}^{I}-\frac{\alpha }{h}\frac{s_{13}^{0}}{s_{11}^{0}}C_{2}^{0}z)e^{\alpha z/h}]{\left(d_{31}^{0}\right)}^{2}+\frac{h}{\alpha }\frac{1}{\lambda_{33}^{0}}[(\frac{\alpha^{2}}{h^{2}}\lambda_{33}^{0}B_{12}^{I}+C_{4}^{0})z-\frac{1}{2}\frac{\alpha }{h}C_{4}^{0}z^{2}\\ \;-(C_{1}^{0}-\frac{h}{\alpha }C_{2}^{0}+C_{2}^{0}z)e^{\alpha z/h}]{\left(d_{33}^{0}\right)}^{2}+s_{13}^{0}[\frac{\alpha }{h}(\frac{\alpha }{h}C_{35}^{\mathrm{II}}-2C_{36}^{\mathrm{II}})z+\frac{\alpha^{2}}{2h^{2}}C_{36}^{\mathrm{II}}z^{2}-2\frac{h^{2}}{\alpha^{2}}\frac{1}{s_{11}^{0}}B_{13}^{I}e^{\alpha z/h}]{\left(d_{15}^{0}\right)}^{2}\\ \;+\{[\frac{\alpha }{h}(B_{2}^{I}+\frac{C_{4}^{0}}{2\lambda_{33}^{0}})x^{2}+\frac{\alpha }{h}(B_{4}^{I}+\frac{C_{8}^{0}}{\lambda_{33}^{0}})x+\frac{\alpha }{h}B_{6}^{I}-\frac{1}{\lambda_{33}^{0}}(-\frac{s_{13}^{0}}{s_{11}^{0}}C_{3}^{0}+\frac{h}{\alpha }\frac{s_{44}^{0}}{s_{11}^{0}}C_{4}^{0}-\frac{h}{\alpha }\frac{\lambda_{11}^{0}}{\lambda_{33}^{0}}C_{4}^{0}-\frac{\alpha }{h}C_{12}^{0}+2\frac{h}{\alpha }\lambda_{11}^{0}B_{2}^{I})\\ \;+s_{13}^{0}G_{2}-\frac{\alpha }{h}s_{13}^{0}F_{2}]z+[s_{13}^{0}(H_{1}-\frac{\alpha }{2h}G_{2})-\frac{1}{\lambda_{33}^{0}}(\frac{s_{13}^{0}}{s_{11}^{0}}\frac{\alpha }{h}C_{3}^{0}-\frac{s_{13}^{0}+2s_{44}^{0}}{2s_{11}^{0}}C_{4}^{0}+\frac{\alpha^{2}}{2h^{2}}C_{12}^{0}-\lambda_{11}^{0}B_{2}^{I})-\frac{\alpha^{2}}{4h^{2}\lambda_{33}^{0}}(2C_{8}^{0}x\\ \;+C_{4}^{0}x^{2})]z^{2}+[s_{13}^{0}(I_{2}-\frac{\alpha }{3h}H_{2})+\frac{\alpha }{h}\frac{1}{6\lambda_{33}^{0}}(\frac{s_{13}^{0}}{s_{11}^{0}}\frac{\alpha }{h}C_{3}^{0}-\frac{2s_{13}^{0}+s_{44}^{0}}{s_{11}^{0}}C_{4}^{0}-\frac{\lambda_{11}^{0}}{\lambda_{33}^{0}}C_{4}^{0})]z^{3}+\frac{\alpha }{4h}(\frac{s_{13}^{0}}{6s_{11}^{0}\lambda_{33}^{0}}\frac{\alpha }{h}C_{4}^{0}\\ \;-s_{13}^{0}I_{2})z^{4}+2\frac{h^{2}}{\alpha^{2}}\frac{s_{13}^{0}}{\lambda_{33}^{0}s_{11}^{0}}(\frac{\alpha }{h}C_{1}^{0}-C_{2}^{0}+\frac{s_{44}^{0}}{2s_{13}^{0}}C_{2}^{0}+\lambda_{11}^{0}\frac{s_{11}^{0}}{s_{13}^{0}}B_{7}+\frac{\alpha }{h}C_{2}^{0}z)e^{\alpha z/h}\}d_{31}^{0}d_{33}^{0}+\frac{s_{13}^{0}}{s_{11}^{0}}[(G_{3}-\frac{\alpha }{h}F_{3})z\\ \;+(H_{3}-\frac{\alpha }{2h}G_{3})z^{2}-\frac{\alpha }{3h}H_{3}z^{3}-(2\frac{h^{2}}{\alpha^{2}}B_{1}^{I}+\frac{1}{\lambda_{33}^{0}}\frac{h^{2}}{\alpha^{2}}C_{2}^{0}+2\frac{1}{\lambda_{33}^{0}}\frac{h^{2}}{\alpha^{2}}\lambda_{11}^{0}B_{13}^{I})e^{\alpha z/h}]d_{31}^{0}d_{15}^{0}\\ \;+\frac{1}{\lambda_{33}^{0}}[(\frac{\alpha }{h}\lambda_{33}^{0}B_{18}^{I}+C_{3}^{0})z+\frac{1}{2}(C_{4}^{0}-\frac{\alpha }{h}C_{3}^{0})z^{2}-\frac{\alpha }{6h}C_{4}^{0}z^{3}+\frac{h^{2}}{\alpha^{2}}(C_{2}^{0}+2\lambda_{11}^{0}B_{13}^{I})e^{\alpha z/h}]d_{33}^{0}d_{15}^{0}+g_{2}(x) \end{array},$$

where $g_{1}(z)$ and $g_{2}(z)$ are unknown functions of *x* and *z*, respectively. Substituting Equations (56) and (57) into the third item of Equation (5) yields,

(58) $$k_{1}z^{2}+k_{2}z+k_{3}e^{\alpha z/h}+k_{4}-\frac{dg_{1}(z)}{dz}=k_{5}x^{3}+k_{6}x^{2}+k_{7}x+\frac{dg_{2}(x)}{dx},$$

where

(59) $$\left\{ \begin{array}{l} k_{1}=-\frac{\alpha^{2}}{2h^{2}}s_{13}^{0}C_{8}^{0}+\frac{\alpha^{2}}{2h^{2}\lambda_{33}^{0}}C_{8}^{0}d_{33}^{0}d_{31}^{0}\\ k_{2}=2s_{13}^{0}\frac{\alpha }{h}C_{8}^{0}-s_{13}^{0}\frac{\alpha^{2}}{h^{2}}C_{7}^{0}+s_{44}^{0}\frac{\alpha }{h}C_{8}^{0}+\frac{\alpha }{h\lambda_{33}^{0}}C_{8}^{0}d_{31}^{0}d_{15}^{0}-(B_{4}^{I}\frac{\alpha }{h}+\frac{\alpha }{h\lambda_{33}^{0}}C_{8}^{0})d_{33}^{0}d_{31}^{0}\\ k_{3}=-B_{3}^{I}d_{31}^{0}d_{15}^{0}-B_{15}^{I}d_{15}^{0}d_{15}^{0}-s_{44}^{0}C_{6}^{0}\\ k_{4}=-B_{4}^{I}d_{31}^{0}d_{15}^{0}-s_{44}^{0}C_{8}^{0}+s_{44}^{0}\frac{\alpha }{h}C_{7}^{0}\\ k_{5}=\frac{1}{6}\frac{\alpha^{2}}{h^{2}}s_{11}^{0}C_{4}^{0}-\frac{1}{3}\frac{\alpha^{2}}{2h^{2}\lambda_{33}^{0}}C_{4}^{0}d_{31}^{0}d_{31}^{0}\\ k_{6}=\frac{1}{2}\frac{\alpha^{2}}{h^{2}}s_{11}^{0}C_{8}^{0}-\frac{1}{2}\frac{\alpha^{2}}{h^{2}\lambda_{33}^{0}}C_{8}^{0}d_{31}^{0}d_{31}^{0}\\ k_{7}=s_{11}^{0}\frac{\alpha^{2}}{h^{2}}C_{12}^{0}+2s_{13}^{0}\frac{\alpha }{h}C_{3}^{0}-s_{44}^{0}C_{4}^{0}-s_{44}^{0}\frac{\alpha }{h}C_{3}^{0}+(-\frac{1}{\lambda_{33}^{0}}C_{4}^{0}-\frac{\alpha }{h}s_{11}^{0}C_{60}^{\mathrm{II}}-2\frac{\lambda_{11}^{0}}{\lambda_{33}^{0}}B_{14}^{I}\\ \;+2B_{2}^{I})d_{31}^{0}d_{15}^{0}-B_{2}^{I}\frac{\alpha }{h}{\left(d_{31}^{0}\right)}^{2}+\frac{\alpha^{2}}{h^{2}}s_{11}^{0}C_{36}^{\mathrm{II}}{\left(d_{15}^{0}\right)}^{2}+(2B_{2}^{I}-C_{48}^{\mathrm{II}}\frac{\alpha }{h}s_{11}^{0})d_{31}^{0}d_{33}^{0} \end{array},\right.$$

By letting

(60) $$k_{1}z^{2}+k_{2}z+k_{3}e^{\alpha z/h}+k_{4}-\frac{dg_{1}(z)}{dz}=k_{5}x^{3}+k_{6}x^{2}+k_{7}x+\frac{dg_{2}(x)}{dx}=v,$$

where v is an undetermined constant, we have

(61) $$\left\{ \begin{array}{l} \frac{dg_{1}(z)}{dz}=k_{1}z^{2}+k_{2}z+k_{3}e^{\alpha z/h}+k_{4}-v\\ \frac{dg_{2}(x)}{dx}=-k_{5}x^{3}-k_{6}x^{2}-k_{7}x+v \end{array}\right..$$

Integrating Equation (61), one has

(62) $$\left\{ \begin{array}{l} g_{1}(z)=\frac{1}{3}k_{1}z^{3}+\frac{1}{2}k_{2}z^{2}+\frac{h}{\alpha }k_{3}e^{\alpha z/h}+k_{4}z-vz+u_{0}\\ g_{2}(x)=-\frac{1}{4}k_{5}x^{4}-\frac{1}{3}k_{6}x^{3}-\frac{1}{2}k_{7}x^{2}+vx+w_{0} \end{array}\right.,$$

where $u_{0}$ and $w_{0}$ are undetermined constants. The undetermined constants v, $u_{0}$, and $w_{0}$ may be determined by Equation (15) (please see Appendix A for details). Substituting Equation (62) into Equations (56) and (57), the final expression of the displacement components may be obtained.

## 4. Results and Discussions

**Figure 2:**
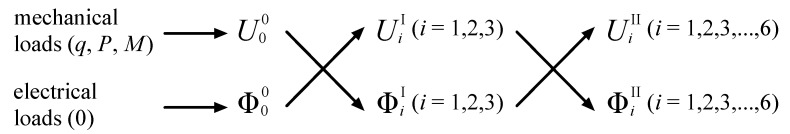
Relationship between the applied loads and the each order perturbation expressions.

In the governing equation, Equation (16), *U* and Φ are coupled with each other. By using M-PPM, Equation (16) is decoupled and simplified, as shown in the decomposed differential equations, i.e., Equation (19), Equations (26)–(28), and Equations (38)–(43). Thus, the perturbation solution of the governing equations can be easily obtained under boundary conditions. From Equations (52) and (54), it can be seen that there are only the first-order perturbation items in the electric field components ($E_{x}$ and $E_{z}$) and electric displacement components ($D_{x}$ and $D_{z}$), which are deduced from the first-order perturbation solutions of the electric potential function, $\Phi_{i}^{I}$ (i=1,2,3). While in the stress components ($\sigma_{x}$, $\sigma_{z}$ and $\tau_{zx}$), strain components ($\varepsilon_{x}$, $\varepsilon_{z}$ and $\gamma_{zx}$), and displacement components (*u* and *w*), there are the zero-order and second-order perturbation items, which are deduced from the zero-order and second-order perturbation solutions of the Airy stress function, $U_{0}^{0}$ and $U_{i}^{\mathrm{II}}$ (i=1,2,3,...,6). This phenomenon can be explained by Figure 2. Figure 2 shows the relationship between the applied mechanical and electrical loads and the each order perturbation expressions of the Airy stress function and electric potential function. 

From Figure 2, it may be seen that the mechanical loads (*q*, *P*, *M*) give rise to $U_{0}^{0}$, $U_{0}^{0}$ gives rise to $\Phi_{i}^{I}$ (i=1,2,3), and then $\Phi_{i}^{I}$ (i=1,2,3) gives rise to $U_{i}^{\mathrm{II}}$ (i=1,2,3,...,6), while $\Phi_{0}^{0}$, $U_{i}^{I}$ (i=1,2,3), and $\Phi_{i}^{\mathrm{II}}$ (i=1,2,3,...,6) have no effect on the stress, strain, displacement, and electric displacement components because the applied electrical loads are 0. Therefore, for the sake of simplification, Equations (17) and (18) may also be written as

(63) $$\Phi =\Phi_{1}^{I}d_{31}^{0}+\Phi_{2}^{I}d_{33}^{0}+\Phi_{3}^{I}d_{15}^{0}$$

and

(64) $$U=U_{0}^{0}+U_{1}^{\mathrm{II}}{\left(d_{31}^{0}\right)}^{2}+U_{2}^{\mathrm{II}}{\left(d_{33}^{0}\right)}^{2}+U_{3}^{\mathrm{II}}{\left(d_{15}^{0}\right)}^{2}+U_{4}^{\mathrm{II}}d_{31}^{0}d_{33}^{0}+U_{5}^{\mathrm{II}}d_{31}^{0}d_{15}^{0}+U_{6}^{\mathrm{II}}d_{33}^{0}d_{15}^{0}.$$

Next, based on the presented perturbation solution, let us consider a functionally graded piezoelectric cantilever beam with l=1 m and h=0.2 m subjected to transverse uniformly distributed loads q=1 N/m^2^ to discuss some related issues. The elastic, piezoelectric, and dielectric constants at z=0 are shown in Table 1 [37].

**Figure 3:**
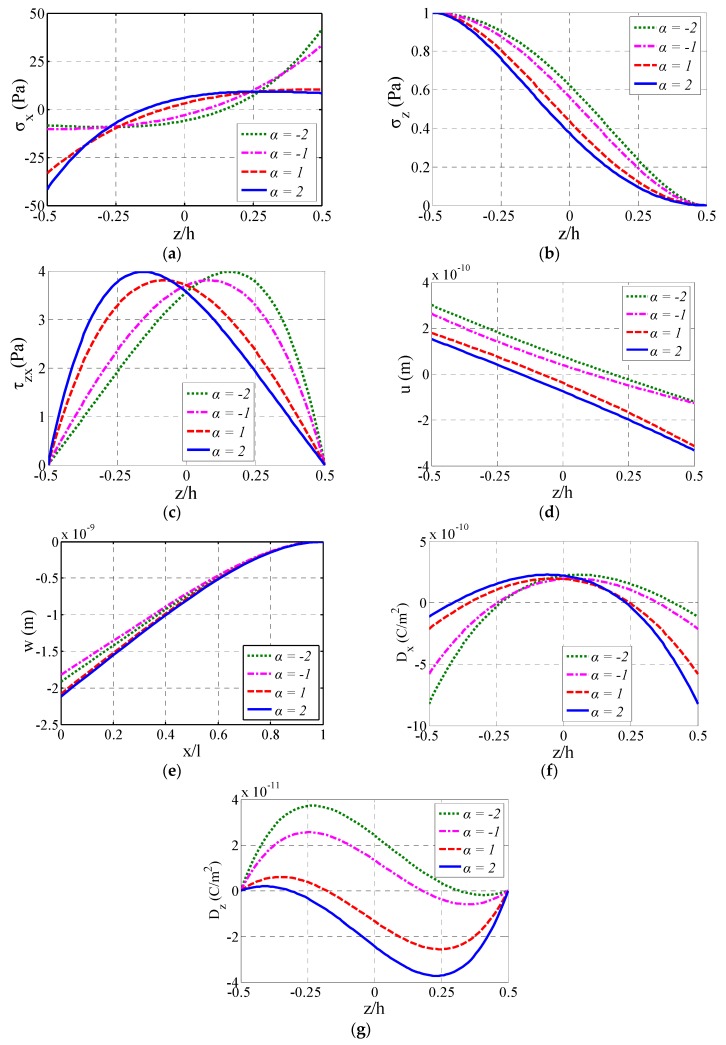
Variation of stresses, displacements, and electric displacements: (**a**) Variation of $\sigma_{x}$ with z/h at x=l/2; (**b**) Variation of $\sigma_{z}$ with z/h at x=l/2; (**c**) Variation of $\tau_{zx}$ with z/h at x=l/2; (**d**) Variation of *u* with z/h at x=l/2; (**e**) Variation of *w* with x/l at z=0; (**f**) Variation of $D_{x}$ with z/h at x=l/2; (**g**) Variation of $D_{z}$ with z/h at x=l/2.

Figure 3 shows the variation of the stress components ($\sigma_{x}$, $\sigma_{z}$ and $\tau_{zx}$), the horizontal displacement (*u*), and the electric displacement components ($D_{x}$ and $D_{z}$) of the cantilever beam at x=l/2 with z/h, and the variation of the vertical deflection *w* at z=0 with x/l, when *α* takes −2, −1, 1, and 2, respectively. 

From Figure 3a,c,d it may be seen that, $\sigma_{x}=0$, u=0, and the maximum shear stress (i.e., $\tau_{zx\max}$) take place at the same z/h when α takes the same value, and this z/h moves toward z/h=−0.5 with the increase of α. For $\sigma_{x}$, when α>0 (or α<0), the maximum compressive stress (or the maximum tensile stress) takes place at z/h=−0.5 (or z/h=0.5), but the maximum tensile stress (or the maximum compressive stress) does not always take place at z/h=0.5 (or z/h=−0.5), especially when the absolute value of *α* (i.e., |α|) is relatively large. In addition, the maximum absolute value of $\sigma_{x}$ (i.e., $\left|\sigma_{x\max}\right|$) and $\tau_{zx\max}$ always take place at the side of αz/h<0 (which means α and *z* are always contrary positive or negative signs since h>0) and increase with the increase of |α|. It is easily seen from Figure 3b that $\sigma_{z}$ decreases with the increase of α. From Figure 3e, it may be seen that, when α<0, *w* decreases with the increase of α, while α>0, the regulation is contrary. Besides, the *w* when α>0 is larger than the one when α<0. From Figure 3f,g, it may be seen that the absolute value of the maximum electric displacements (i.e., $\left|D_{x\max}\right|$ and $\left|D_{z\max}\right|$) always takes place at the side of αz/h>0 (which means α and *z* are always identically positive or negative signs since h>0), because the piezoelectric coefficient $d_{ij}=d_{ij}^{0}e^{\alpha z/h}$ at the side of αz/h>0 is larger than the $d_{ij}$ at the side of αz/h<0. In addition, $\left|D_{x\max}\right|$ and $\left|D_{z\max}\right|$ also increase with the increase of |α|.

## 5. Concluding Remarks

In this study, by extending the traditional single-parameter and biparametric perturbation method to the multi-parameter perturbation method, we solved the problem of a functionally graded piezoelectric cantilever beam under the combined action of uniformly distributed loads, concentrated load, and bending moment. The following main conclusions can be drawn.

(i) By selecting the piezoelectric coefficients as perturbation parameters, the multi-parameter perturbation method can be used to decouple and simplify the governing equations of the functionally graded piezoelectric cantilever beam.

(ii) The expansion expression of the Airy stress function and electric potential function with respect to the perturbation parameters, i.e., Equations (17) and (18), can be simplified to Equations (63) and (64), when only mechanical loads are applied on the functionally graded piezoelectric cantilever beam. 

(iii) The $\left|\sigma_{x\max}\right|$ and $\tau_{zx\max}$ always take place at the side of αz/h<0, and the $\left|D_{x\max}\right|$ and $\left|D_{z\max}\right|$ always take place at the side of αz/h>0, but they all increase with the increase of |α|.

It should be pointed out that the analytical results found in the sample example should be validated by comparison with other numerical methods (e.g., Finite Element results) and/or experimental tests. Besides, the multi-parameter perturbation method may also be applicable to the problem of other functionally graded piezoelectric structures under electrical loads or electro-mechanical loads. In these cases, different boundary conditions concerning mechanical or electrical properties will inevitably introduce some new influences on the final results. Due to the fact that the analytical expressions obtained are expressed in terms of the piezoelectric coefficients, we can see clearly how the piezoelectric effects influence the behavior of the functionally graded piezoelectric structural element, which is exactly the benefit of parameter-based perturbation solutions. Therefore, as far as the practical application of the work is concerned, the results obtained in this study may serve as a theoretical guide for the design of smart structures with functionally graded piezoelectric structural elements. 

Finally, it should be noted here that in our multi-parameter perturbation method, the parameters are not dependent on each other, thus leading to a large number of independent perturbation equations. However, in the literature, there exists an alternative and much more efficient method [38,39,40,41,42], in which all the parameters (irrespective of their number) are perturbed together along straight lines in the parameter space, thus formally re-conducting the multi-parameter case to that of a single parameter. At the end of the procedure, however, the parameters can be varied independently, since the exploring straight line can be freely chosen. It can be expected that this procedure can be used to solve this kind of problem effectively, and possibly be contrasted to the results obtained in our work. We will study these interesting issues in the future.

## Figures and Tables

**Table 1:** Elastic, piezoelectric, and dielectric constants of the cantilever beam at z=0.

Elastic Constant (10^−12^ m^2^/N)	Piezoelectric Constant (10^−12^ C/N)	Dielectric Constant (10^−8^ F/m)
$s_{11}^{0}$ $s_{13}^{0}$ $s_{33}^{0}$ $s_{44}^{0}$	$d_{31}^{0}$ $d_{33}^{0}$ $d_{15}^{0}$	$\lambda_{11}^{0}$ $\lambda_{33}^{0}$
12.4 −5.52 16.1 39.1	−135 300 525	1.301 1.151

## Appendix A

(A1) $$\left\{ \begin{array}{l} C_{1}^{0}=\frac{q(2e^{-\alpha }-\alpha^{2}-2\alpha -2)}{2(e^{\alpha }+e^{-\alpha }-\alpha^{2}-2)},C_{2}^{0}=\frac{q\alpha^{2}}{h(e^{\alpha }+e^{-\alpha }-\alpha^{2}-2)}\\ C_{3}^{0}=\frac{q(2e^{\alpha }+\alpha e^{\alpha }+\alpha -2)}{2e^{\alpha /2}(e^{\alpha }+e^{-\alpha }-\alpha^{2}-2)},C_{4}^{0}=\frac{q\alpha (e^{\alpha }-1)}{he^{\alpha /2}(e^{\alpha }+e^{-\alpha }-\alpha^{2}-2)} \end{array}\right.,$$

(A2) $$\left\{ \begin{array}{l} C_{6}^{0}=-\frac{P\alpha^{2}}{bh(e^{\alpha }+e^{-\alpha }-\alpha^{2}-2)},C_{7}^{0}=-\frac{P(2e^{\alpha }+\alpha e^{\alpha }+\alpha -2)}{be^{-\alpha /2}(e^{\alpha }+e^{-\alpha }-\alpha^{2}-2)}\\ C_{8}^{0}=-\frac{P\alpha (e^{\alpha }-1)}{bhe^{-\alpha /2}(e^{\alpha }+e^{-\alpha }-\alpha^{2}-2)} \end{array}\right.,$$

(A3) $$\left\{ \begin{array}{l} C_{11}^{0}=\{-\frac{1}{s_{11}^{0}}[\frac{1}{2}(s_{13}^{0}C_{1}^{0}+\frac{h}{\alpha }s_{44}^{0}C_{2}^{0})N_{1}+\frac{1}{3}s_{13}^{0}C_{2}^{0}N_{2}](N_{3}-\frac{\alpha }{h}N_{4})-\frac{1}{2}(\frac{s_{13}^{0}}{s_{11}^{0}}C_{3}^{0}-\frac{s_{44}^{0}}{s_{11}^{0}}\frac{h}{\alpha }C_{4}^{0})N_{5}(N_{3}-\frac{\alpha }{h}N_{4})\\ +(\frac{1}{2}\frac{\alpha }{h}\frac{s_{13}^{0}}{s_{11}^{0}}C_{3}^{0}-\frac{1}{2}\frac{s_{44}^{0}}{s_{11}^{0}}C_{4}^{0}-\frac{1}{3}\frac{s_{13}^{0}}{s_{11}^{0}}C_{4}^{0})N_{6}(N_{3}-\frac{\alpha }{h}N_{4})+\frac{1}{6}\frac{\alpha }{h}\frac{s_{13}^{0}}{s_{11}^{0}}C_{4}^{0}(N_{3}-\frac{\alpha }{h}N_{4})N_{7}-\frac{M}{b}(N_{3}-\frac{\alpha }{h}N_{4})\\ -[\frac{1}{2s_{11}^{0}}\frac{\alpha }{h}(2s_{13}^{0}C_{1}^{0}h+2s_{44}^{0}\frac{h}{\alpha }C_{2}^{0}h+s_{13}^{0}C_{2}^{0}N_{1})N_{5}+\frac{\alpha }{h}(\frac{s_{13}^{0}}{s_{11}^{0}}C_{3}^{0}-\frac{s_{44}^{0}}{s_{11}^{0}}\frac{h}{\alpha }C_{4}^{0})N_{5}N_{4}-\frac{1}{2}\frac{\alpha }{h}(\frac{\alpha }{h}\frac{s_{13}^{0}}{s_{11}^{0}}C_{3}^{0}-\frac{s_{13}^{0}}{s_{11}^{0}}C_{4}^{0}\\ -\frac{s_{44}^{0}}{s_{11}^{0}}C_{4}^{0})N_{5}N_{5}-\frac{\alpha^{2}}{h^{2}}\frac{s_{13}^{0}}{6s_{11}^{0}}C_{4}^{0}N_{5}N_{6}]\}/[(N_{3}+\frac{\alpha }{h}N_{4})(N_{3}-\frac{\alpha }{h}N_{4})+\frac{\alpha^{2}}{h^{2}}N_{5}N_{3}]\\ C_{12}^{0}=\frac{1}{\left(N_{3}-\frac{\alpha }{h}N_{4}\right)}[\frac{\alpha }{h}C_{11}^{0}N_{3}+\frac{1}{2s_{11}^{0}}(2s_{13}^{0}C_{1}^{0}h+2s_{44}^{0}\frac{h}{\alpha }C_{2}^{0}h+s_{13}^{0}C_{2}^{0}N_{1})\\ +(\frac{s_{13}^{0}}{s_{11}^{0}}C_{3}^{0}-\frac{s_{44}^{0}}{s_{11}^{0}}\frac{h}{\alpha }C_{4}^{0})N_{4}-\frac{1}{2}(\frac{\alpha }{h}\frac{s_{13}^{0}}{s_{11}^{0}}C_{3}^{0}-\frac{s_{13}^{0}}{s_{11}^{0}}C_{4}^{0}-\frac{s_{44}^{0}}{s_{11}^{0}}C_{4}^{0})N_{5}-\frac{\alpha }{h}\frac{s_{13}^{0}}{6s_{11}^{0}}C_{4}^{0}N_{6}] \end{array},\right.$$

where

(A4) $$\left\{ \begin{array}{l} N_{1}=0,N_{2}=\frac{h^{3}}{4},N_{3}=e^{-\frac{\alpha }{2}}-e^{\frac{\alpha }{2}},N_{4}=\frac{h}{2}(e^{-\frac{\alpha }{2}}+e^{\frac{\alpha }{2}})\\ N_{5}=\frac{h^{2}}{4}(e^{-\frac{\alpha }{2}}-e^{\frac{\alpha }{2}}),N_{6}=\frac{h^{3}}{8}(e^{-\frac{\alpha }{2}}+e^{\frac{\alpha }{2}}),N_{7}=\frac{h^{4}}{16}(e^{-\frac{\alpha }{2}}-e^{\frac{\alpha }{2}}) \end{array}\right..$$

(A5) $$B_{1}^{0}=B_{2}^{0}=B_{3}^{0}=B_{4}^{0}=B_{6}^{0}=0.$$

(A6) $$\left\{ \begin{array}{l} C_{1}^{I}=C_{2}^{I}=C_{3}^{I}=C_{4}^{I}=C_{6}^{I}=C_{7}^{I}=C_{8}^{I}=C_{11}^{I}=C_{12}^{I}=0\\ C_{13}^{I}=C_{14}^{I}=C_{15}^{I}=C_{16}^{I}=C_{18}^{I}=C_{19}^{I}=C_{20}^{I}=C_{23}^{I}=C_{24}^{I}=0\\ C_{25}^{I}=C_{26}^{I}=C_{27}^{I}=C_{28}^{I}=C_{30}^{I}=C_{31}^{I}=C_{32}^{I}=C_{35}^{I}=C_{36}^{I}=0 \end{array}\right..$$

(A7) $$\left\{ \begin{array}{l} B_{1}^{I}=\frac{1}{\lambda_{33}^{0}}\alpha (C_{4}^{0}-\frac{\alpha }{h}C_{3}^{0})/N_{3}\\ B_{2}^{I}=(C_{4}^{0}-\frac{\alpha }{h}C_{3}^{0})/2\lambda_{33}^{0}\\ B_{3}^{I}=-\frac{\alpha^{2}}{4h^{2}\lambda_{33}^{0}}[h^{2}C_{8}^{0}-2\alpha B_{4}^{I}]/N_{3}\\ B_{4}^{I}=(C_{8}^{0}-\frac{\alpha }{h}C_{7}^{0})/\lambda_{33}^{0}\\ B_{6}^{I}=[-2\frac{h^{2}}{\alpha^{2}}\lambda_{11}^{0}B_{1}^{I}e^{\frac{\alpha }{2}}+(-\frac{\lambda_{11}^{0}}{\lambda_{33}^{0}}\frac{h^{2}}{\alpha^{2}}C_{4}^{0}+C_{12}^{0}+2\frac{h^{2}}{\alpha^{2}}\lambda_{11}^{0}B_{2}^{I}-C_{11}^{0}\frac{\alpha }{h})\\ \;-\frac{h}{\alpha }\lambda_{11}^{0}B_{2}^{I}h+\frac{1}{8}\frac{\lambda_{11}^{0}}{\lambda_{33}^{0}}C_{4}^{0}h^{2}]/\lambda_{33}^{0} \end{array},\right.$$

(A8) $$\left\{ \begin{array}{l} B_{7}^{I}=B_{8}^{I}=B_{9}^{I}=B_{10}^{I}=0\\ B_{12}^{I}=-\frac{h}{\alpha }\frac{1}{\lambda_{33}^{0}}(C_{3}^{0}+\frac{h}{\alpha }C_{4}^{0}) \end{array}\right.,$$

(A9) $$\left\{ \begin{array}{l} B_{13}^{I}=\frac{1}{2\lambda_{11}^{0}}[\alpha (C_{4}^{0}-\frac{\alpha }{h}C_{3}^{0})-C_{2}^{0}N_{3}]/N_{3}\\ B_{14}^{I}=B_{16}^{I}=0\\ B_{15}^{I}=\frac{1}{\lambda_{11}^{0}}[\alpha (C_{8}^{0}-\frac{\alpha }{h}C_{7}^{0})-C_{6}^{0}N_{3}]/N_{3}\\ B_{18}^{I}=\frac{1}{\lambda_{33}^{0}N_{3}}(C_{3}^{0}N_{4}+\frac{1}{2}C_{4}^{0}N_{5}-C_{3}^{0}\frac{h}{\alpha }N_{3}-C_{4}^{0}\frac{h}{\alpha }N_{4}) \end{array}\right..$$

(A10) $$\left\{ \begin{array}{l} C_{1}^{\mathrm{II}}=0{,}_{}^{}C_{2}^{\mathrm{II}}=-2\frac{1}{s_{11}^{0}}B_{1}^{I},C_{3}^{\mathrm{II}}=-2\frac{B_{2}^{I}}{s_{11}^{0}},C_{4}^{\mathrm{II}}=\frac{\alpha }{h}\frac{C_{4}^{0}}{s_{11}^{0}\lambda_{33}^{0}}\\ C_{6}^{\mathrm{II}}=-\frac{1}{s_{11}^{0}}B_{3}^{I},C_{7}^{\mathrm{II}}=-\frac{B_{4}^{I}}{s_{11}^{0}}{,}_{}^{}C_{8}^{\mathrm{II}}=\frac{\alpha }{h}\frac{C_{8}^{0}}{s_{11}^{0}\lambda_{33}^{0}}\\ C_{11}^{\mathrm{II}}=(-2J_{1}h-G_{1}N_{4}-H_{1}N_{5}-I_{1}N_{6})/N_{3}-\frac{1}{s_{11}^{0}}B_{6}^{I}\\ C_{12}^{\mathrm{II}}=[N_{3}(-K_{1}N_{2}+H_{1}N_{6}+I_{1}N_{7})-(\frac{h}{\alpha }N_{3}+N_{4})(2J_{1}h+H_{1}N_{5}+I_{1}N_{6})]/[(\frac{h}{\alpha }N_{3}+N_{4})N_{4}\\ -(\frac{h^{2}}{\alpha^{2}}N_{3}+\frac{h}{\alpha }N_{4}+N_{5})N_{3}]-\frac{h}{\alpha }\frac{1}{\lambda_{33}^{0}}\frac{1}{s_{11}^{0}}(-\frac{s_{13}^{0}}{s_{11}^{0}}\frac{\alpha }{h}C_{3}^{0}+\frac{s_{44}^{0}}{s_{11}^{0}}C_{4}^{0}-\frac{\lambda_{11}^{0}}{\lambda_{33}^{0}}C_{4}^{0}-\frac{\alpha^{2}}{h^{2}}C_{12}^{0}+2\lambda_{11}^{0}B_{2}^{I}) \end{array},\right.$$

where

(A11) $$\left\{ \begin{array}{l} F_{1}=C_{11}^{\mathrm{II}}+\frac{1}{s_{11}^{0}}B_{6}^{I}\\ G_{1}=\frac{h}{\alpha }\frac{1}{\lambda_{33}^{0}}\frac{1}{s_{11}^{0}}(-3\frac{s_{13}^{0}}{s_{11}^{0}}\frac{\alpha }{h}C_{3}^{0}+\frac{3s_{44}^{0}}{s_{11}^{0}}C_{4}^{0}-\frac{\lambda_{11}^{0}}{\lambda_{33}^{0}}C_{4}^{0}-\frac{\alpha^{2}}{h^{2}}C_{12}^{0}+2\lambda_{11}^{0}B_{2}^{I})+C_{12}^{\mathrm{II}}\\ H_{1}=\frac{1}{2\lambda_{33}^{0}}\frac{1}{s_{11}^{0}}(-\frac{s_{13}^{0}}{s_{11}^{0}}\frac{\alpha }{h}C_{3}^{0}-\frac{s_{13}^{0}-s_{44}^{0}}{s_{11}^{0}}C_{4}^{0}-\frac{\lambda_{11}^{0}}{\lambda_{33}^{0}}C_{4}^{0})-\frac{1}{s_{11}^{0}}(\frac{s_{13}^{0}}{s_{11}^{0}}B_{2}^{I}+\frac{s_{13}^{0}}{2}C_{3}^{\mathrm{II}}+\frac{1}{2\lambda_{33}^{0}}\frac{s_{44}^{0}}{s_{11}^{0}}C_{4}^{0}-\frac{h}{\alpha }\frac{s_{44}^{0}}{2}C_{4}^{\mathrm{II}})\\ I_{1}=\frac{s_{13}^{0}}{6s_{11}^{0}\lambda_{33}^{0}}\frac{1}{s_{11}^{0}}\frac{\alpha }{h}C_{4}^{0}+\frac{s_{13}^{0}}{6s_{11}^{0}}(\frac{\alpha }{h}\frac{1}{s_{11}^{0}}\frac{1}{\lambda_{33}^{0}}C_{4}^{0}-C_{4}^{\mathrm{II}})\\ J_{1}=-\frac{1}{2s_{11}^{0}}\frac{1}{\lambda_{33}^{0}s_{11}^{0}}\frac{h}{\alpha }(s_{13}^{0}\frac{\alpha }{h}C_{1}^{0}+s_{44}^{0}C_{2}^{0}+2\lambda_{11}^{0}s_{11}^{0}B_{1}^{I})\\ K_{1}=\frac{1}{s_{11}^{0}}\frac{s_{13}^{0}}{3\lambda_{33}^{0}s_{11}^{0}}C_{2}^{0} \end{array}.\right.$$

(A12) $$C_{13}^{\mathrm{II}}=C_{14}^{\mathrm{II}}=C_{15}^{\mathrm{II}}=C_{16}^{\mathrm{II}}=C_{18}^{\mathrm{II}}=C_{19}^{\mathrm{II}}=C_{20}^{\mathrm{II}}=C_{23}^{\mathrm{II}}=C_{24}^{\mathrm{II}}=0,$$

(A13) $$\left\{ \begin{array}{l} C_{25}^{\mathrm{II}}=C_{26}^{\mathrm{II}}=C_{27}^{\mathrm{II}}=C_{28}^{\mathrm{II}}=C_{30}^{\mathrm{II}}=C_{31}^{\mathrm{II}}=C_{32}^{\mathrm{II}}=0\\ C_{35}^{\mathrm{II}}=\frac{h}{\alpha }(-2\frac{h}{\alpha }\frac{1}{s_{11}^{0}}B_{13}^{I}h-\frac{\alpha }{h}C_{36}^{\mathrm{II}}N_{4}+C_{36}^{\mathrm{II}}N_{3})/N_{3}\\ C_{36}^{\mathrm{II}}=2\frac{h}{\alpha }\frac{1}{s_{11}^{0}}B_{13}^{I}h(N_{3}+\frac{\alpha }{h}N_{4})/(N_{3}^{2}-\frac{\alpha^{2}}{h^{2}}N_{4}^{2}+\frac{\alpha^{2}}{h^{2}}N_{5}N_{3}) \end{array}\right.,$$

(A14) $$\left\{ \begin{array}{l} C_{37}^{\mathrm{II}}=C_{38}^{\mathrm{II}}=C_{39}^{\mathrm{II}}=C_{40}^{\mathrm{II}}=C_{42}^{\mathrm{II}}=C_{43}^{\mathrm{II}}=C_{44}^{\mathrm{II}}=0\\ C_{47}^{\mathrm{II}}=-(\frac{1}{\lambda_{33}^{0}}\frac{1}{s_{11}^{0}}C_{1}^{0}h+G_{2}N_{4}+H_{2}N_{5}+I_{2}N_{6})/N_{3}+\frac{1}{\lambda_{33}^{0}}\frac{1}{s_{11}^{0}}\frac{h}{\alpha }(C_{3}^{0}+\frac{h}{\alpha }C_{4}^{0})\\ C_{48}^{\mathrm{II}}=[(\frac{1}{3}\frac{1}{\lambda_{33}^{0}}\frac{1}{s_{11}^{0}}C_{2}^{0}N_{2}+H_{2}N_{6}+I_{2}N_{7})N_{3}-(N_{4}+\frac{h}{\alpha }N_{3})(\frac{1}{\lambda_{33}^{0}}\frac{1}{s_{11}^{0}}C_{1}^{0}h+H_{2}N_{5}\\ +I_{2}N_{6})]/[(N_{4}+\frac{h}{\alpha }N_{3})N_{4}-(\frac{h^{2}}{\alpha^{2}}N_{3}+\frac{h}{\alpha }N_{4}+N_{5})N_{3}]+\frac{1}{\lambda_{33}^{0}}\frac{1}{s_{11}^{0}}\frac{h}{\alpha }C_{4}^{0} \end{array},\right.$$

where

(A15) $$\left\{ \begin{array}{l} F_{2}=C_{47}^{\mathrm{II}}-\frac{1}{\lambda_{33}^{0}}\frac{1}{s_{11}^{0}}\frac{h}{\alpha }(C_{3}^{0}+\frac{h}{\alpha }C_{4}^{0}){,}_{}^{}G_{2}=C_{48}^{\mathrm{II}}-\frac{1}{\lambda_{33}^{0}}\frac{1}{s_{11}^{0}}\frac{h}{\alpha }C_{4}^{0}\\ H_{2}=\frac{1}{s_{11}^{0}}B_{2}^{I}+\frac{\alpha }{2h}\frac{s_{13}^{0}}{s_{11}^{0}}C_{39}^{\mathrm{II}}+\frac{s_{13}^{0}+s_{14}^{0}}{2s_{11}^{0}}C_{40}^{\mathrm{II}}{,}_{}^{}I_{2}=-\frac{\alpha }{6h}(\frac{1}{\lambda_{33}^{0}}\frac{1}{s_{11}^{0}}C_{4}^{0}-\frac{s_{13}^{0}}{s_{11}^{0}}C_{40}^{\mathrm{II}}) \end{array}\right..$$

(A16) $$\left\{ \begin{array}{l} C_{49}^{\mathrm{II}}=C_{50}^{\mathrm{II}}=C_{51}^{\mathrm{II}}=C_{52}^{\mathrm{II}}=C_{54}^{\mathrm{II}}=C_{55}^{\mathrm{II}}=C_{56}^{\mathrm{II}}=0\\ C_{59}^{\mathrm{II}}=[(2\frac{h}{\alpha }B_{1}^{I}h+\frac{1}{\lambda_{33}^{0}}\frac{h}{\alpha }(C_{2}^{0}+2\lambda_{11}^{0}B_{13}^{I})h-G_{3}N_{4}-H_{3}N_{5})/N_{3}-B_{18}^{I}]/s_{11}^{0}\\ C_{60}^{\mathrm{II}}=\frac{1}{s_{11}^{0}}(G_{3}+\frac{1}{\lambda_{33}^{0}}C_{3}^{0}-2\frac{h}{\alpha }\frac{\lambda_{11}^{0}}{\lambda_{33}^{0}}B_{14}^{I}) \end{array}\right.,$$

where

(A17) $$F_{3}=B_{18}^{I}+s_{11}^{0}C_{59}^{\mathrm{II}},G_{3}=s_{11}^{0}C_{60}^{\mathrm{II}}+2\frac{h}{\alpha }\frac{\lambda_{11}^{0}}{\lambda_{33}^{0}}B_{14}^{I}-\frac{1}{\lambda_{33}^{0}}C_{3}^{0},H_{3}=-\frac{1}{\lambda_{33}^{0}}C_{4}^{0}.$$

(A18) $$C_{61}^{\mathrm{II}}=C_{62}^{\mathrm{II}}=C_{63}^{\mathrm{II}}=C_{64}^{\mathrm{II}}=C_{66}^{\mathrm{II}}=C_{67}^{\mathrm{II}}=C_{68}^{\mathrm{II}}=C_{71}^{\mathrm{II}}=C_{72}^{\mathrm{II}}=0.$$

(A19) $$\left\{ \begin{array}{l} B_{1}^{\mathrm{II}}=B_{2}^{\mathrm{II}}=B_{3}^{\mathrm{II}}=B_{4}^{\mathrm{II}}=B_{6}^{\mathrm{II}}=0\\ B_{7}^{\mathrm{II}}=B_{8}^{\mathrm{II}}=B_{9}^{\mathrm{II}}=B_{10}^{\mathrm{II}}=B_{12}^{\mathrm{II}}=0\\ B_{13}^{\mathrm{II}}=B_{14}^{\mathrm{II}}=B_{15}^{\mathrm{II}}=B_{16}^{\mathrm{II}}=B_{18}^{\mathrm{II}}=0\\ B_{19}^{\mathrm{II}}=B_{20}^{\mathrm{II}}=B_{21}^{\mathrm{II}}=B_{22}^{\mathrm{II}}=B_{24}^{\mathrm{II}}=0\\ B_{25}^{\mathrm{II}}=B_{26}^{\mathrm{II}}=B_{27}^{\mathrm{II}}=B_{28}^{\mathrm{II}}=B_{30}^{\mathrm{II}}=0\\ B_{31}^{\mathrm{II}}=B_{32}^{\mathrm{II}}=B_{33}^{\mathrm{II}}=B_{34}^{\mathrm{II}}=B_{36}^{\mathrm{II}}=0 \end{array}\right..$$

The solving process of undetermined constants v, $u_{0}$, and $w_{0}$: substituting Equations (49) and (50) into Equation (15), we have

(A20) $$\begin{array}{l} {u|}_{\begin{array}{l} z=0\\ x=l \end{array}}=-\frac{l^{3}}{3}\frac{\alpha }{h}s_{11}^{0}C_{4}^{0}+\frac{l^{3}}{6}\frac{\alpha^{2}}{h^{2}}s_{11}^{0}C_{3}^{0}+\frac{l^{2}}{2}\frac{\alpha^{2}}{h^{2}}s_{11}^{0}C_{7}^{0}-l^{2}\frac{\alpha }{h}s_{11}^{0}C_{8}^{0}-2\frac{\alpha }{h}s_{11}^{0}C_{12}^{0}l+\frac{\alpha^{2}}{h^{2}}s_{11}^{0}C_{11}^{0}l\\ +\frac{h}{\alpha }s_{44}^{0}C_{4}^{0}l-\frac{h}{\alpha }s_{44}^{0}C_{2}^{0}l+(s_{11}^{0}G_{2}-\frac{\alpha }{h}s_{11}^{0}F_{2}+\frac{1}{\lambda_{33}^{0}}\frac{h}{\alpha }C_{4}^{0}+\frac{\alpha }{h}B_{12}^{I})ld_{31}^{0}d_{33}^{0}+(G_{3}-\frac{\alpha }{h}F_{3}+\frac{1}{\lambda_{33}^{0}}C_{3}^{0}\\ +\frac{\alpha }{h}B_{18}^{I}-2\frac{h}{\alpha }B_{1}^{I})ld_{31}^{0}d_{15}^{0}+(\frac{\alpha^{2}}{h^{2}}s_{11}^{0}C_{35}^{\mathrm{II}}-2\frac{\alpha }{h}s_{11}^{0}C_{36}^{\mathrm{II}}-2\frac{h}{\alpha }B_{13}^{I})l{\left(d_{15}^{0}\right)}^{2}+[-\frac{l^{3}}{3}(-\frac{\alpha }{h}B_{2}^{I}\\ -\frac{\alpha }{2h\lambda_{33}^{0}}C_{4}^{0})-\frac{l^{2}}{2}(-\frac{\alpha }{h}B_{4}^{I}-\frac{\alpha }{h\lambda_{33}^{0}}C_{8}^{0})+\frac{\alpha }{h}B_{6}^{I}l-\frac{h}{\alpha }\frac{1}{\lambda_{33}^{0}}(-\frac{\alpha }{h}\frac{s_{13}^{0}}{s_{11}^{0}}C_{3}^{0}+\frac{s_{44}^{0}}{s_{11}^{0}}C_{4}^{0}-\frac{\lambda_{11}^{0}}{\lambda_{33}^{0}}C_{4}^{0}\\ -\frac{\alpha^{2}}{h^{2}}C_{12}^{0}+2\lambda_{11}^{0}B_{2}^{I})l+(s_{11}^{0}G_{1}-\frac{\alpha }{h}s_{11}^{0}F_{1})l]{\left(d_{31}^{0}\right)}^{2}+\frac{h}{\alpha }k_{3}+u_{0}=0 \end{array},$$

(A21) $$\begin{array}{l} {w|}_{\begin{array}{l} z=0\\ x=l \end{array}}=\frac{h}{\alpha }(s_{33}^{0}C_{1}^{0}-s_{13}^{0}\frac{s_{13}^{0}}{s_{11}^{0}}C_{1}^{0}-s_{44}^{0}\frac{s_{13}^{0}}{s_{11}^{0}}\frac{h}{\alpha }C_{2}^{0})-(s_{33}^{0}C_{2}^{0}-s_{13}^{0}\frac{s_{13}^{0}}{s_{11}^{0}}C_{2}^{0})\frac{h^{2}}{\alpha^{2}}+[\frac{1}{\lambda_{33}^{0}s_{11}^{0}}\frac{h^{2}}{\alpha^{2}}(s_{13}^{0}\frac{\alpha }{h}C_{1}^{0}\\ +s_{44}^{0}C_{2}^{0}+2\lambda_{11}^{0}s_{11}^{0}B_{7})-\frac{2s_{13}^{0}}{\lambda_{33}^{0}s_{11}^{0}}\frac{h^{2}}{\alpha^{2}}C_{2}^{0}+\frac{h}{\alpha }\frac{1}{\lambda_{33}^{0}}\frac{s_{13}^{0}}{s_{11}^{0}}C_{1}^{0}]d_{31}^{0}d_{33}^{0}+\frac{1}{\lambda_{33}^{0}}\frac{h^{2}}{\alpha^{2}}(C_{2}^{0}+2\lambda_{11}^{0}B_{13}^{I})d_{33}^{0}d_{15}^{0}\\ -(2\frac{s_{13}^{0}}{s_{11}^{0}}\frac{h^{2}}{\alpha^{2}}B_{1}^{I}+\frac{1}{\lambda_{33}^{0}}\frac{s_{13}^{0}}{s_{11}^{0}}\frac{h^{2}}{\alpha^{2}}C_{2}^{0}+2\frac{1}{\lambda_{33}^{0}}\frac{s_{13}^{0}}{s_{11}^{0}}\frac{h^{2}}{\alpha^{2}}\lambda_{11}^{0}B_{13}^{I})d_{31}^{0}d_{15}^{0}+(\frac{1}{\lambda_{33}^{0}}\frac{h^{2}}{\alpha^{2}}C_{2}^{0}-\frac{1}{\lambda_{33}^{0}}\frac{h}{\alpha }C_{1}^{0}){\left(d_{33}^{0}\right)}^{2}\\ -2\frac{h^{2}}{\alpha^{2}}\frac{1}{s_{11}^{0}}B_{13}^{I}s_{13}^{0}{\left(d_{15}^{0}\right)}^{2}+[\frac{s_{13}^{0}}{s_{11}^{0}}\frac{s_{13}^{0}}{\lambda_{33}^{0}s_{11}^{0}}\frac{h^{2}}{\alpha^{2}}C_{2}^{0}-\frac{s_{13}^{0}}{s_{11}^{0}}\frac{1}{\lambda_{33}^{0}}\frac{h^{2}}{\alpha^{2}}(\frac{s_{13}^{0}}{s_{11}^{0}}\frac{\alpha }{h}C_{1}^{0}+\frac{s_{44}^{0}}{s_{11}^{0}}C_{2}^{0}+2\lambda_{11}^{0}B_{1}^{I})]{\left(d_{31}^{0}\right)}^{2}\\ -\frac{1}{4}k_{5}l^{4}-\frac{1}{3}k_{6}l^{3}-\frac{1}{2}k_{7}l^{2}+vl+w_{0}=0 \end{array}$$

and

(A22) $${\frac{\partial w}{\partial x}|}_{\begin{array}{l} z=0\\ x=l \end{array}}=-k_{5}l^{3}-k_{6}l^{2}-k_{7}l+v=0.$$

From Equations (A20)–(A22), it can be obtained that

(A23) $$v=k_{5}l^{3}+k_{6}l^{2}+k_{7}l,$$

(A24) $$\begin{array}{l} u_{0}=\frac{l^{3}}{3}\frac{\alpha }{h}s_{11}^{0}C_{4}^{0}-\frac{l^{3}}{6}\frac{\alpha^{2}}{h^{2}}s_{11}^{0}C_{3}^{0}-\frac{l^{2}}{2}\frac{\alpha^{2}}{h^{2}}s_{11}^{0}C_{7}^{0}+l^{2}\frac{\alpha }{h}s_{11}^{0}C_{8}^{0}+2\frac{\alpha }{h}s_{11}^{0}C_{12}^{0}l-\frac{\alpha^{2}}{h^{2}}s_{11}^{0}C_{11}^{0}l-\frac{h}{\alpha }s_{44}^{0}C_{4}^{0}l\\ \;+\frac{h}{\alpha }s_{44}^{0}C_{2}^{0}l-(s_{11}^{0}G_{2}-\frac{\alpha }{h}s_{11}^{0}F_{2}+\frac{1}{\lambda_{33}^{0}}\frac{h}{\alpha }C_{4}^{0}+\frac{\alpha }{h}B_{12}^{I})ld_{31}^{0}d_{33}^{0}-(G_{3}-\frac{\alpha }{h}F_{3}+\frac{1}{\lambda_{33}^{0}}C_{3}^{0}+\frac{\alpha }{h}B_{18}^{I}\\ \;-2\frac{h}{\alpha }B_{1}^{I})ld_{31}^{0}d_{15}^{0}-(\frac{\alpha^{2}}{h^{2}}s_{11}^{0}C_{35}^{\mathrm{II}}-2\frac{\alpha }{h}s_{11}^{0}C_{36}^{\mathrm{II}}-2\frac{h}{\alpha }B_{13}^{I})l{\left(d_{15}^{0}\right)}^{2}-[-\frac{l^{3}}{3}(-\frac{\alpha }{h}B_{2}^{I}-\frac{\alpha }{2h\lambda_{33}^{0}}C_{4}^{0})\\ \;-\frac{l^{2}}{2}(-\frac{\alpha }{h}B_{4}^{I}-\frac{\alpha }{h\lambda_{33}^{0}}C_{8}^{0})+\frac{\alpha }{h}B_{6}^{I}l-\frac{h}{\alpha }\frac{1}{\lambda_{33}^{0}}(-\frac{\alpha }{h}\frac{s_{13}^{0}}{s_{11}^{0}}C_{3}^{0}+\frac{s_{44}^{0}}{s_{11}^{0}}C_{4}^{0}-\frac{\lambda_{11}^{0}}{\lambda_{33}^{0}}C_{4}^{0}-\frac{\alpha^{2}}{h^{2}}C_{12}^{0}+2\lambda_{11}^{0}B_{2}^{I})l\\ \;+(s_{11}^{0}G_{1}-\frac{\alpha }{h}s_{11}^{0}F_{1})l]{\left(d_{31}^{0}\right)}^{2}-\frac{h}{\alpha }k_{3} \end{array}$$

and

(A25) $$\begin{array}{l} w_{0}=-\frac{h}{\alpha }(s_{33}^{0}C_{1}^{0}-s_{13}^{0}\frac{s_{13}^{0}}{s_{11}^{0}}C_{1}^{0}-s_{44}^{0}\frac{s_{13}^{0}}{s_{11}^{0}}\frac{h}{\alpha }C_{2}^{0})+(s_{33}^{0}C_{2}^{0}-s_{13}^{0}\frac{s_{13}^{0}}{s_{11}^{0}}C_{2}^{0})\frac{h^{2}}{\alpha^{2}}-[\frac{1}{\lambda_{33}^{0}s_{11}^{0}}\frac{h^{2}}{\alpha^{2}}(s_{13}^{0}\frac{\alpha }{h}C_{1}^{0}\\ \;+s_{44}^{0}C_{2}^{0}+2\lambda_{11}^{0}s_{11}^{0}B_{7})-\frac{2s_{13}^{0}}{\lambda_{33}^{0}s_{11}^{0}}\frac{h^{2}}{\alpha^{2}}C_{2}^{0}+\frac{h}{\alpha }\frac{1}{\lambda_{33}^{0}}\frac{s_{13}^{0}}{s_{11}^{0}}C_{1}^{0}]d_{31}^{0}d_{33}^{0}-\frac{1}{\lambda_{33}^{0}}\frac{h^{2}}{\alpha^{2}}(C_{2}^{0}+2\lambda_{11}^{0}B_{13}^{I})d_{33}^{0}d_{15}^{0}\\ \;+(2\frac{s_{13}^{0}}{s_{11}^{0}}\frac{h^{2}}{\alpha^{2}}B_{1}^{I}+\frac{1}{\lambda_{33}^{0}}\frac{s_{13}^{0}}{s_{11}^{0}}\frac{h^{2}}{\alpha^{2}}C_{2}^{0}+2\frac{1}{\lambda_{33}^{0}}\frac{s_{13}^{0}}{s_{11}^{0}}\frac{h^{2}}{\alpha^{2}}\lambda_{11}^{0}B_{13}^{I})d_{31}^{0}d_{15}^{0}-[\frac{s_{13}^{0}}{s_{11}^{0}}\frac{s_{13}^{0}}{\lambda_{33}^{0}s_{11}^{0}}\frac{h^{2}}{\alpha^{2}}C_{2}^{0}\\ \;-\frac{s_{13}^{0}}{s_{11}^{0}}\frac{1}{\lambda_{33}^{0}}\frac{h^{2}}{\alpha^{2}}(\frac{s_{13}^{0}}{s_{11}^{0}}\frac{\alpha }{h}C_{1}^{0}+\frac{s_{44}^{0}}{s_{11}^{0}}C_{2}^{0}+2\lambda_{11}^{0}B_{1}^{I})]{\left(d_{31}^{0}\right)}^{2}-(\frac{1}{\lambda_{33}^{0}}\frac{h^{2}}{\alpha^{2}}C_{2}^{0}-\frac{1}{\lambda_{33}^{0}}\frac{h}{\alpha }C_{1}^{0}){\left(d_{33}^{0}\right)}^{2}\\ \;+2\frac{h^{2}}{\alpha^{2}}\frac{1}{s_{11}^{0}}B_{13}^{I}s_{13}^{0}{\left(d_{15}^{0}\right)}^{2}-\frac{3}{4}k_{5}l^{4}-\frac{2}{3}k_{6}l^{3}-\frac{1}{2}k_{7}l^{2} \end{array}.$$
